# Metabolomic Profiling of Brain Protective Effect of Edaravone on Cerebral Ischemia-Reperfusion Injury in Mice

**DOI:** 10.3389/fphar.2022.814942

**Published:** 2022-02-14

**Authors:** Hui-fen Ma, Fan Zheng, Lin-jie Su, Da-wei Zhang, Yi-ning Liu, Fang Li, Yuan-yuan Zhang, Shuai-shuai Gong, Jun-ping Kou

**Affiliations:** State Key Laboratory of Natural Medicines, Jiangsu Key Laboratory of TCM Evaluation and Translational Research, Department of Pharmacology of Chinese Materia Medica, School of Traditional Pharmacy, China Pharmaceutical University, Nanjing, China

**Keywords:** metabolomic, edaravone, ischemia stroke, taurine, endothelial cells

## Abstract

Edaravone (EDA) injection has been extensively applied in clinics for treating stroke. Nevertheless, the metabolite signatures and underlying mechanisms associated with EDA remain unclear, which deserve further elucidation for improving the accurate usage of EDA. Ischemia stroke was simulated by intraluminal occlusion of the right middle cerebral artery for 1 h, followed by reperfusion for 24 h in mice. Brain infarct size, neurological deficits, and lactate dehydrogenase (LDH) levels were improved by EDA. Significantly differential metabolites were screened with untargeted metabolomics by cross-comparisons with pre- and posttreatment of EDA under cerebral ischemia/reperfusion (I/R) injury. The possibly involved pathways, such as valine, leucine, and isoleucine biosynthesis, and phenylalanine, taurine, and hypotaurine metabolisms, were enriched with differential metabolites and relevant regulatory enzymes, respectively. The network of differential metabolites was constructed for the integral exhibition of metabolic characteristics. Targeted analysis of taurine, an important metabolic marker, was performed for further validation. The level of taurine decreased in the MCAO/R group and increased in the EDA group. The inhibition of EDA on cerebral endothelial cell apoptosis was confirmed by TdT-mediated dUTP nick-end labeling (TUNEL) stain. Cysteine sulfinic acid decarboxylase (CSAD), the rate-limiting enzyme of taurine generation, significantly increased along with inhibiting endothelial cell apoptosis after treatment of EDA. Thus, CSAD, as the possible new therapeutic target of EDA, was selected and validated by Western blot and immunofluorescence. Together, this study provided the metabolite signatures and identified CSAD as an unrecognized therapeutic intervention for EDA in the treatment of ischemic stroke *via* inhibiting brain endothelial cell apoptosis.

## Introduction

Stroke, one of the neurovascular diseases, is the leading cause of disability and death globally, resulting from an increasing burden of vascular risk factors. Ischemic stroke is the most common type, accounting for 70% of all strokes (Benjamin et al., 2019; Montaner et al., 2020). Although the precise mechanism underlying ischemic injury has not been fully elucidated, vascular pathology has been reported the most common cause. As a part of the vascular pathologies, endothelial cell (EC) death could affect the surrounding cellular environment, which made it a potential target mechanism for the treatment and prevention of stroke, and ECs line the entire microvasculature and are also important for maintaining normal brain function. Therefore, it is necessary to choose the appropriate drugs for ischemic stroke.

Edaravone injection (EDA), as a commonly neurovascular protective agent, has been widely used in patients with acute ischemic stroke owing to its scavenging effect on oxygen-free radical and neurovascular protective effects (Kikuchi et al., 2013). It has been proven that EDA attenuates the Ca^2+^-induced swelling of mitochondria and inhibits neuron apoptosis by decreasing the expression of Fas-associated death domain protein, death-associated protein, and caspase-8 immunoreactivity in the middle cerebral artery occlusion (MCAO) model (Zhang et al., 2005). EDA could suppress the response to endoplasmic reticulum stress and subsequent apoptotic signaling in hypoxic/ischemic injury and exhibit neuroprotective effects *via* its antioxidant actions, such as suppression of lipid peroxidation and oxidant-induced DNA damage (Amemiya et al., 2005; Yung et al., 2007). In addition, EDA also could inhibit vascular endothelial growth factor (VEGF) expression, aquaporin-4 expression, nuclear factor-κB (NF-κB), inducible nitric oxide synthase (iNOS), cytokines, cyclooxygenase-2, reactive oxygen species (ROS) generation, and ROS-induced inflammatory reactions in stroke mice and patients (Kikuchi et al., 2013). However, few of the literature comprehensively elucidate action characteristics of EDA; thus, further studies are still needed.

Metabolites are small molecules (typically <1.5 kDa), including lipids, amino acids, carbohydrates, and nucleotides, that could reflect the downstream function of the gene, protein expression, and environmental changes, such as drug intake; as a result, metabolome could provide information about related mechanisms (Shah et al., 2012). What is more, disease-specific metabolites can be biomarkers for the diagnosis of diseases and provide reference for the precise use of drugs in the clinic. The functional characteristics of Huang-Lian-Jie-Du decoction and gross saponins of *Tribulus terrestris* fruit were elucidated for ischemic stroke with metabolomics (Fu et al., 2019; Wang et al., 2019). By contrast, the value of metabolites of EDA for stroke has not been systematically studied. Therefore, the metabolomics was selected to investigate the potential mechanism of EDA.

Herein, we intended to discover the therapeutic mechanism of EDA for stroke as comprehensively as possible according to the metabolite variation characteristics. In this study, untargeted metabolic profiling was applied to examine the serum and urine metabolic signature of EDA for improving stroke. The metabolic network was constructed with differential metabolites. Finally, we performed targeted metabolic profiling and verified the potential therapeutic targets.

## Materials and Methods

### Chemicals and Reagents

The standard compounds of taurine and caffeic acid were obtained from Shanghai Yuanye Bio-technology Co., Ltd. (Shanghai, China). Deionized water used in the experiment was supplied by a Milli-Q Academic ultrapure water system (Milford, Millipore, United States). Acetonitrile and methanol were obtained from Merck (Chromatographic, Germany); formic acid was obtained from Tedia (Chromatographic, United States). Edaravone injection was obtained from China National Medicines Guorui pharmaceutical Co., Ltd. (Anhui, China; lot number: 2005018). The lactate dehydrogenase (LDH) assay kit was purchased from Nanjing Jiancheng Bioengineering Institute (Nanjing, China), and cysteine sulfinic acid decarboxylase (CSAD) was obtained from Abcam (Cambridge, England).

### Animals and Middle Cerebral Artery Occlusion/Reperfusion (MCAO/R) Model

Adult male specified-pathogen-free (SPF) C57BL/6J mice weighing 18–22 g were obtained from the Experimental Animal Research Centre of Yangzhou University (Yangzhou, China; certificate no SCXK 2017–0007). All experimental protocols were performed according to the National Institutes of Health (NIH) guidelines and the research was approved by the Institutional Animal Care and Use Committee of the Animal Ethics Committee of the School of Chinese Materia Medica, China Pharmaceutical University. All mice were housed with a 12:12 h light–dark cycle at 23 ± 1°C. Prior to experiments, mice were split randomly into three groups: sham, MCAO/R, and MCAOR + EDA. Stroke was induced by the MCAO/R model in mice as reported previously (Cao et al., 2016). In addition, the right middle cerebral artery was occluded with a blunt-tip 6-0 nylon monofilament for 1 h. Then the animals were reperfused by the careful withdrawal of the filament. Sham-operated control mice underwent the same surgical procedures except for the occlusion by nylon monofilament. EDA was administrated intraperitoneally to mice with 3 mg/kg (refer to the clinical dose) after 1 h of ischemia, the remaining model mice were given an equal volume of normal saline. Neurological function was evaluated at 24 h after reperfusion. Neurological deficit was graded on a score of 0–4 as previously reported (Cao et al., 2016) with slight modifications, as follows: 0, no observable deficit; 1, forelimb flexion and preference to walk in one direction; 2, unable to walk straight or to turn in both directions, circling to the affected side when held by the tail on the bench; 3, circling on the spot and walking circling; and 4, no spontaneous locomotor activity or barrel rolling, upon stimulation circling.

### Hematoxylin and Eosin (H&E) Staining

H&E staining was used for histomorphological analysis. In short, brain slices were put into hematoxylin and eosin solution, redehydrated in gradient ethanol solution again, treated with dimethylbenzene, and covered with coverslips. The pathological images were scanned with a digital pathological section scanner (Hamamatsu, Japan) and analyzed with NDPView2 software.

### TTC Staining

After ischemia/reperfusion (I/R), mice were euthanized and perfused by normal saline. Then, the whole brains were taken out, frozen at −20° followed by cutting into 1 mm thick slices rapidly. These brain slices were incubated in 1% TTC for 10 min at 37°C. The infarcted areas were analyzed with ImageJ software (NIH, Bethesda, MD).

### Transmission Electron Microscopy

After I/R, mice were euthanized and perfused by normal saline followed by perfusion with the fixative (2% glutaraldehyde and 2% lanthanum nitrate in 0.1M sodium cacodylate pH 7.4–7.5) at room temperature, as previously described (Wang Q. et al., 2007). 1 mm^3^ sample obtained from the region encompassing ischemic infarction of removed brains was kept in the same fixative overnight at 4°C. The samples were postfixed in 1% osmium tetroxide for 1 h followed by embedding in Epon 812. After polymerization, three blocks were randomly selected from each brain sample. An Ultratome (Nova, LKB, Bromma, Sweden) was used for cutting ultrathin sections. Then, ultrath insections were mounted on mesh grids (6–8 sections/grid) and stained with uranyl acetate and lead citrate. Finally, the prepared samples were examined under a transmission electron microscope (JEOL Ltd., Tokyo, Japan).

### Untargeted Metabolomics Analysis

#### Sample Pretreatment

Serum and urine of mice were collected after 24 h reperfusion. After standing for about 60 min, the blood was centrifuged with 3,500 r/min for 10 min at 15°C. The obtained serum samples were sub-packed and stored at −80°C until the analysis. Urine samples were collected at 4°C and kept at −80 °C until the analysis. 200 μl of serum and urine were used for untargeted metabolomics analysis and 600 μl of methanol was added into samples for precipitating protein. Samples were subsequently centrifuged (13,000 rpm, 15 min) at 4°C followed by swirling 60 s. The supernatant was transferred to a tube and dried under a gentle stream of nitrogen at room temperature. Then, the residue was dissolved with 200 μl methanol and centrifuged (13,000 rpm, 15 min) at 4°C for further analysis.

#### HPLC-Q-TOF/MS Analysis

The detection of metabolites in urine and serum samples was performed on an Agilent Technologies 6540 Accurate-Mass Q-TOF LC/MS (United States) with electrospray ionization (ESI) source and the data were collected by a mass hunter workstation. The eluant A and B were deionized water (0.1% formic acid) and acetonitrile (0.1% formic acid), respectively. Serum analyses were achieved on a SynergiTM Fusion-RP C18 column (50 × 2 mm i.d., 2.5 μm) with a gradient elution program: 0–5 min, 5–5% B; 5–10 min, 5–30% B; 10–15 min, 30–60% B; 15–20 min, 60–70% B; 20–22 min, 70–80% B; 22–25 min, 80–95% B; 25–30 min, 95–95% B. Urine analyses were achieved on a TSK-GEL Amide-80 column (150 × 2.0 mm i.d., 5 μm) with a gradient elution program: 0–7 min, 90–90% B; 7–9 min, 90–75% B; 9–11 min, 75–75% B; 11–13 min, 75–50% B; 13–20 min, 50–50% B. Both of the flow rates were set at 0.2 ml/min with the injection volume of 10 μl. The Q-TOF/MS operating parameters were set as follows: fragment voltage, 120 V; nebulizer gas, 35 psig; capillary voltage, 4000 V; drying gas flow rate, 9 L/min; temperature, 325°C; detection range, m/z 50–1,500 in full scan mass spectra. The MS data acquisition was carried out in positive and negative ionization modes.

#### Validation of System Stability

The repeatability and robustness of the experiment were validated with the pooled quality control sample (QC) (Peron et al., 2020). The QC sample was prepared to mix equal volumes (30 μl) of each test sample, and treated with the same method as the test samples. QC samples were randomly injected throughout the sequence list.

#### Data Analysis of Metabolomics Strategies

Before multivariate analysis, the data format (.mzdata) files obtained by MassHunter Workstation Software (version B.06.00, Agilent Technologies) were processed by XCMS software performing on the R+ package (R Foundation for Statistical Computing, Vienna, Austria), and the data pretreatment procedures include non-linear retention time alignment, peak discrimination, filtering, alignment, and matching. All detected peaks were tabulated with tR-m/z pairs and outputted for statistical analyses. In order to screen the significant compounds that were responsible for the difference between model and model + EDA, metabolomic strategies were subsequently used to dispose the data. Principal component analysis (PCA), orthogonal partial least square discriminant analysis (OPLS-DA), volcano Plot, and heatmap developed by Metaboanalyst (https://www.metaboanalyst.ca/) were adopted to do the preliminary screening. PCA is a multivariate technique which can select the typical variables from a data table by several linear transformations, and OPLS-DA is a supervised machine learning model. The online database including HMDB (http://www.hmdb.ca/), METLIN (http://metlin.scripps.edu/), and MassBank (http://www.massbank.jp/) was performed to identify the potential metabolites by matching with the message of ion fragments.

### Targeted Analysis for Taurine by HPLC-QQQ-MS/MS

Targeted analysis was performed on a triple quadrupole tandem high-performance liquid chromatography-mass spectrometry (HPLC-QQQ-MS/MS) system (Agilent, 6465) with caffeic acid as the internal standard. Chromatographic separation was performed on a TSK-GEL Amide-80 column (150 × 2.0 mm i.d., 5 μm) with a gradient elution program: 0–1 min, 75–75% B; 1–2 min, 75–60% B; 2–3 min, 60–60% B; 3–5 min, 60–50% B. The mobile phase system consists of deionized water containing 0.1% formic acid (A) and acetonitrile containing 0.1% formic acid (B) at a flow rate of 0.2 ml/min. Multiple reaction monitoring transitions in the negative mode were performed at m/z124→79.9 for the target analyte taurine and m/z 179→135 for the internal standard compound. MS parameters for the LC-MS/MS system, including the fragment and voltage collision energy of taurine and internal standard were 110, 21, and 90 V, 17 V, respectively.

### Western Blot Analysis

The RIPA buffer supplemented with protease inhibitor cocktail was adopted for lysing ischemic penumbra of the brain tissues, and obtained samples were used for Western blotting as described previously (Zhai et al., 2017). Protein concentration of tissues was determined by Bicinchoninic Acid (BCA) Protein Assay Kit (Biyuntian Biotech. Co., Ltd., China) after centrifuging (12,000 rpm, 10 min, 4°C). The supernatant was diluted by loading buffer to 1 μg/μl followed by heating at 100°C for 5 min. Equal protein amounts of different groups were electrophoresed on SDS-PAGE gels and transferred to a polyvinylidene fluoride (PVDF) membrane. Then, the obtained PVDF membrane was blocked with 5% BSA solution for 2 h and incubated with specific primary antibodies overnight at 4°C followed by suitable secondary antibodies at room temperature for 2 h. Protein signals were detected with the ECL plus system and imaged by the gel imaging system (BioRad, Hercules, CA, United States). The protein levels were calculated by protein signals to correlative GAPDH or β-actin.

### Immunofluorescence Staining

After perfusion with PBS and 4% paraformaldehyde, brain tissues were picked up and put into 4% paraformaldehyde. After 24 h, brain tissues were dehydrated with 40% sucrose for 5 days, embedded in OTC, and frozen at −80°C. Brain tissues were sectioned into slices of 10 μm thickness with a cryotome (Leica, Mannheim, Germany). Brain sections were fixed in 4% paraformaldehyde, permeabilized with 0.3% Triton X-100 in PBS, blocked with 5% bovine serum albumin, and incubated with specific primary antibodies overnight at 4°C. The next day, tissue sections were incubated with appropriate fluorescence-conjugated secondary antibodies at room temperature, and the cell nucleus was stained with DAPI. The immunofluorescence TUNEL assay was performed according to the instructions of the manufacturer. Fluorescent images were observed by confocal laser scanning microscopy (CLSM, LSM700, Zeiss, Germany).

### Statistical Analysis

Student’s t-test and one-way analysis of variance (ANOVA) followed by Dunnett’s *post hoc* test operating on the GraphPad Prism 8.0 (Graph Pad Software, La Jolla, CA, United States) were used for analyzing two group comparisons and multiple comparisons, respectively. Differences were considered significant at *p* < .05.

## Results

### EDA Effectively Ameliorated Brain Ischemia Reperfusion Injury in Mice

**FIGURE 1:**
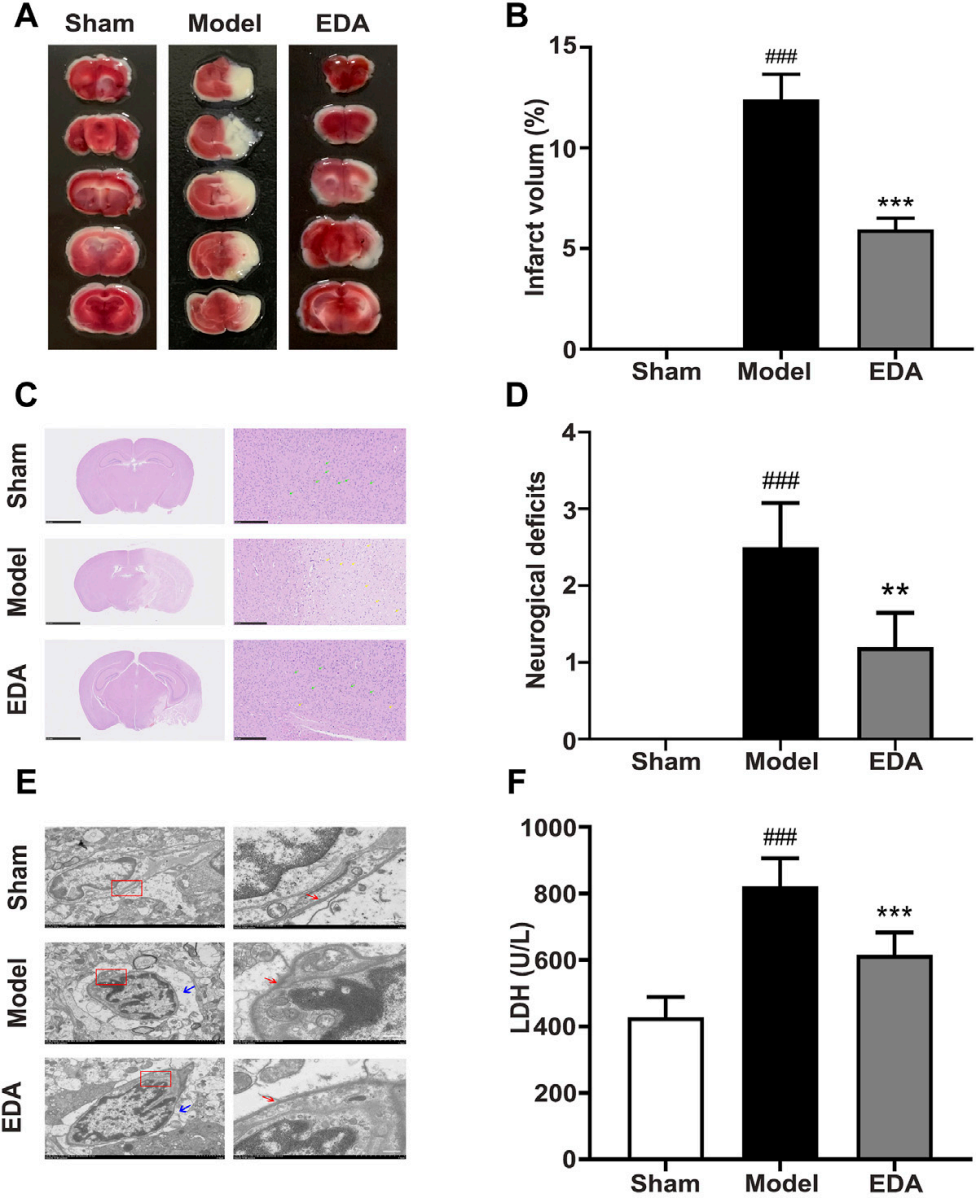
EDA protects against cerebral I/R injury and endothelial injury. Mice were subjected to 1 h of ischemia, followed by 24 h of reperfusion. EDA (3 mg/kg) was administered intraperitoneally after ischemia. **(A)** Representative TTC-stained brain sections. **(B)** Quantitative analysis of infarct volume. **(C)** Stained H&E sections of mice brains. Shrunken cells with pyknotic nuclei are indicated with yellow arrows, while intact cells are indicated with green arrows. **(D)** Neurological deficit scores in different groups. **(E)** The structure and morphology of cerebral microvascular endothelial cells in different groups were examined by electron microscopy. Red arrow: brain microvascular endothelial cell membrane. Blue arrow: the degree of edema around brain microvascular endothelial cells. **(F)** LDH activity. All data are presented as the means ± SEM, *n* = 6. Scale bar = 50 μm. ^#^ *p* < .05, ^##^ *p* < .01, ^###^ *p* < .001, *vs*. Sham group, *p < .05, **p < .01, ***p < .001, *vs*. MCAO/R group.

The results of TTC staining demonstrated the marked infarct area of the brain appeared after cerebral I/R and could be reduced by EDA (Figures 1A,B). H&E staining of brain sections showed that cerebral I/R induced cell loss and numerous vacuolated spaces, whereas EDA ameliorated such histopathological damage, as shown in Figure 1C. Additionally, the neurobehavioral deficits could be improved by EDA administration compared with the model group (Figure 1D). The electron microscope was applied for observing the morphology of endothelium, the key elements of the blood–brain barrier. Obviously, the endothelial cells were destroyed after cerebral I/R and improved by EDA (Figure 1E). The morphology of cerebral microvascular endothelial cells in MCAO/R mice changed. Additionally, the cell membrane integrity was also destroyed in MCAO/R mice. These injuries of microvascular endothelial cells could be improved by EDA. Besides, the level of LDH in serum increased in MCAO/R mice and could be significantly inhibited by EDA (Figure 1F). Taken together, EDA effectively alleviated the brain injury and inflammation in MCAO/R mice.

### Multivariate Statistical Analysis of Metabolites in Urine and Serum Samples

Analytical stability was validated by contrasting the difference in retention time of the QC samples. The overlapped total ion chromatograms of QC samples showed that retention time deviation was acceptable (Supplementary Figures 1A–D). Three ions were randomly chosen from QC samples including serum-positive, serum-negative, urine-positive, and urine-negative to evaluate the system reproducibility in the metabolomic raw data acquisition throughout the whole experiment. The relative standard deviations (RSD) of the retention times and corresponding peak areas of the 3 selected ions in the QC samples were 0.59–2.54 and 1.14%–3.78%, as shown in Table 1. The results proved that the repeatability and stability of the HPLC-Q-TOF/MS system were reliable.

**TABLE 1:** Relative standard deviation (RSD%) of retention time and peak area in QC samples.

Sample	Model	m/z	Retention time (RSD%)	Peak area (RSD%)
Serum	Positive	203.0541	1.34	2.01
		274.2751	0.95	1.93
		675.6783	1.12	3.56
	Negative	215.0316	1.27	3.78
		809.2477	2.08	2.69
		279.2312	2.54	2.17
Urine	Positive	174.1122	1.83	2.98
		114.0654	0.81	1.74
		263.1456	1.96	3.14
	Negative	172.9869	1.53	1.14
		208.0667	0.59	1.22
		195.0460	1.21	1.65

**FIGURE 2:**
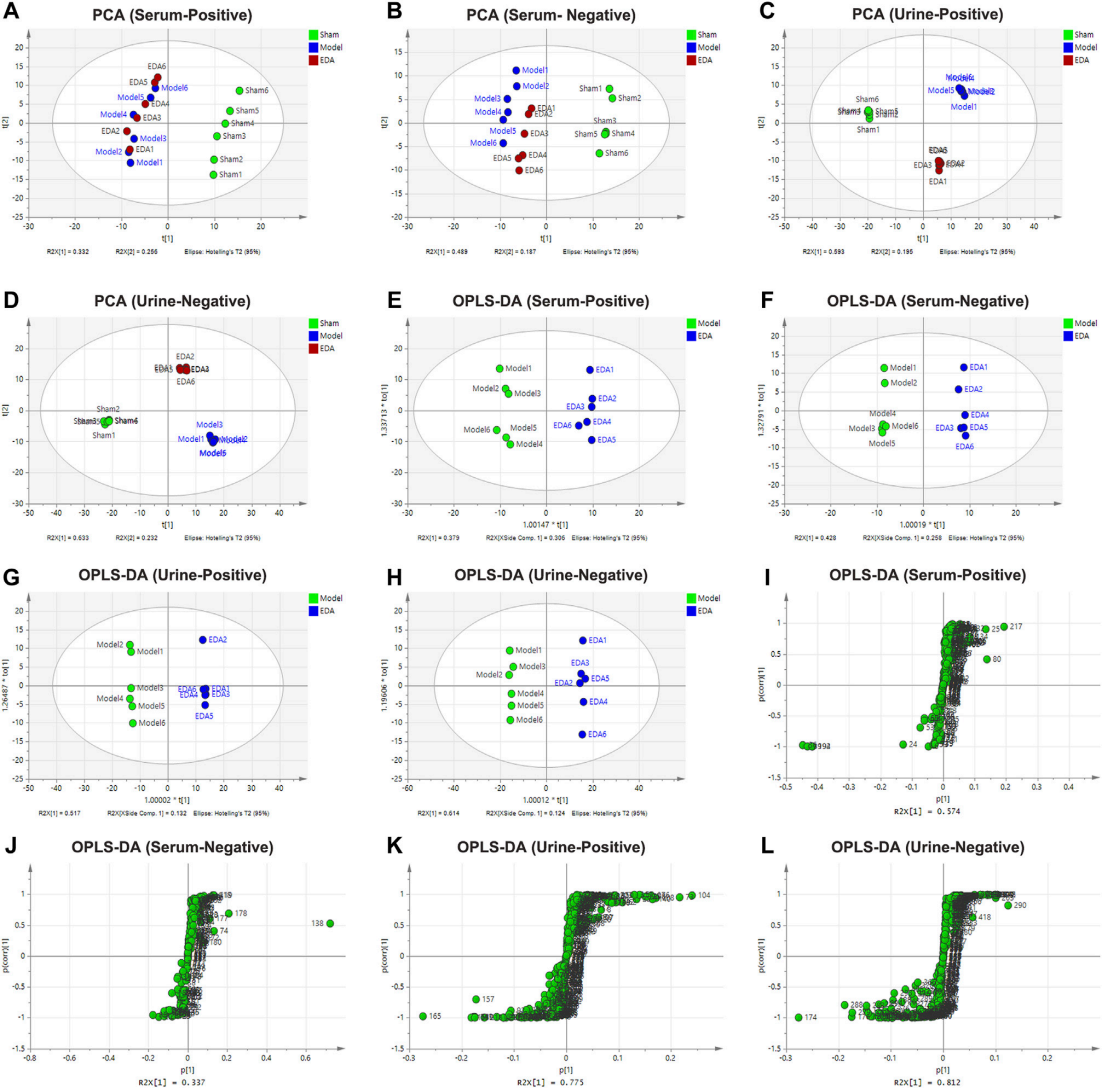
PCA, OPLS-DA score plot, and S-plot of sham, model, and EDA group based on HPLC-Q-TOF/MS system for serum and urine analysis. **(A–D)** PCA score plot of model and EDA group. **(E–H)** OPLS-DA score plot of model and EDA group. **(I–L)** S-plot of model and EDA group.

**FIGURE 3:**
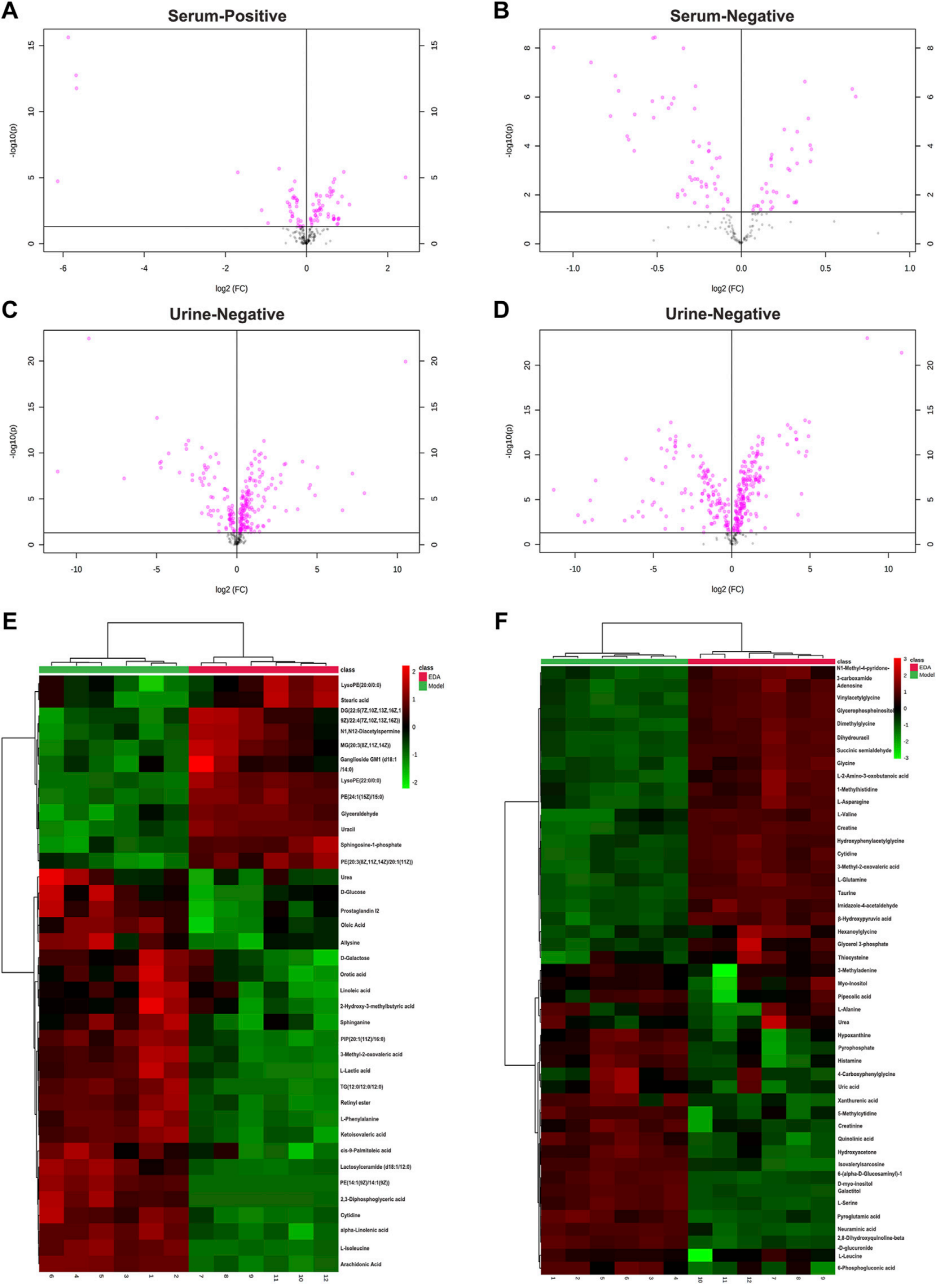
Volcano plot and heatmap of the differential endogenous metabolites between the model and EDA group in serum and urine. **(A–D)** Volcano plot of model and EDA group. **(E)** Heatmap of the differential endogenous metabolites between the model and EDA group in serum. **(F)** Heatmap of the differential endogenous metabolites between the model and EDA group in urine. Red represented the metabolites in high abundance; green represented the metabolites in low abundance.

PCA was applied to perform unsupervised data analysis on Sham, MCAO/R, and EDA groups, and these groups could be easily distinguished from each other (Figures 2A–D). The phenomenon of the EDA group closing to the sham group compared with the MCAO/R group showed the improvement of EDA on brain injury. To screen the influential compounds that caused the difference between EDA and the model group, OPLS-DA was applied to classify the different samples and select the differential compounds from obtained data. Figures 2E–H suggested that the metabolic profiles in the EDA group were significantly different from those in the MCAO/R group in both urine and serum samples, and the ions of variable importance parameters (VIP > 1) were obtained. S-plot was applied to show those changed ions which significantly contributed to the classification between EDA and MCAO/R group (Figures 2I–L). Depending on VIP > 1 and *p*-value (*p* < .05) acquired through two-tailed Student’s t-test and showed in volcano plot (Figures 3A–D), the variables can be selected for further screening. According to the above screening procedures, the ions were screened and the metabolites were identified, which were considered as potential biomarkers listed in Supplementary Table S1. Comparing EDA and MCAO/R groups, 51 and 56 differential metabolites were identified in serum and urine, respectively. The hierarchical clustering heatmap exhibited the change of metabolites more intuitively (Figures 3E,F). The heatmaps showed that EDA and MCAO/R could be grouped into two parts according to the identified metabolites. The above data exposed that numerous metabolites changed by EDA. Among these metabolites, taurine with 30.795 of fold change showed the greatest change.

### Enrichment Analysis of Metabolic Pathway and Regulatory Enzymes Changed by EDA

**FIGURE 4:**
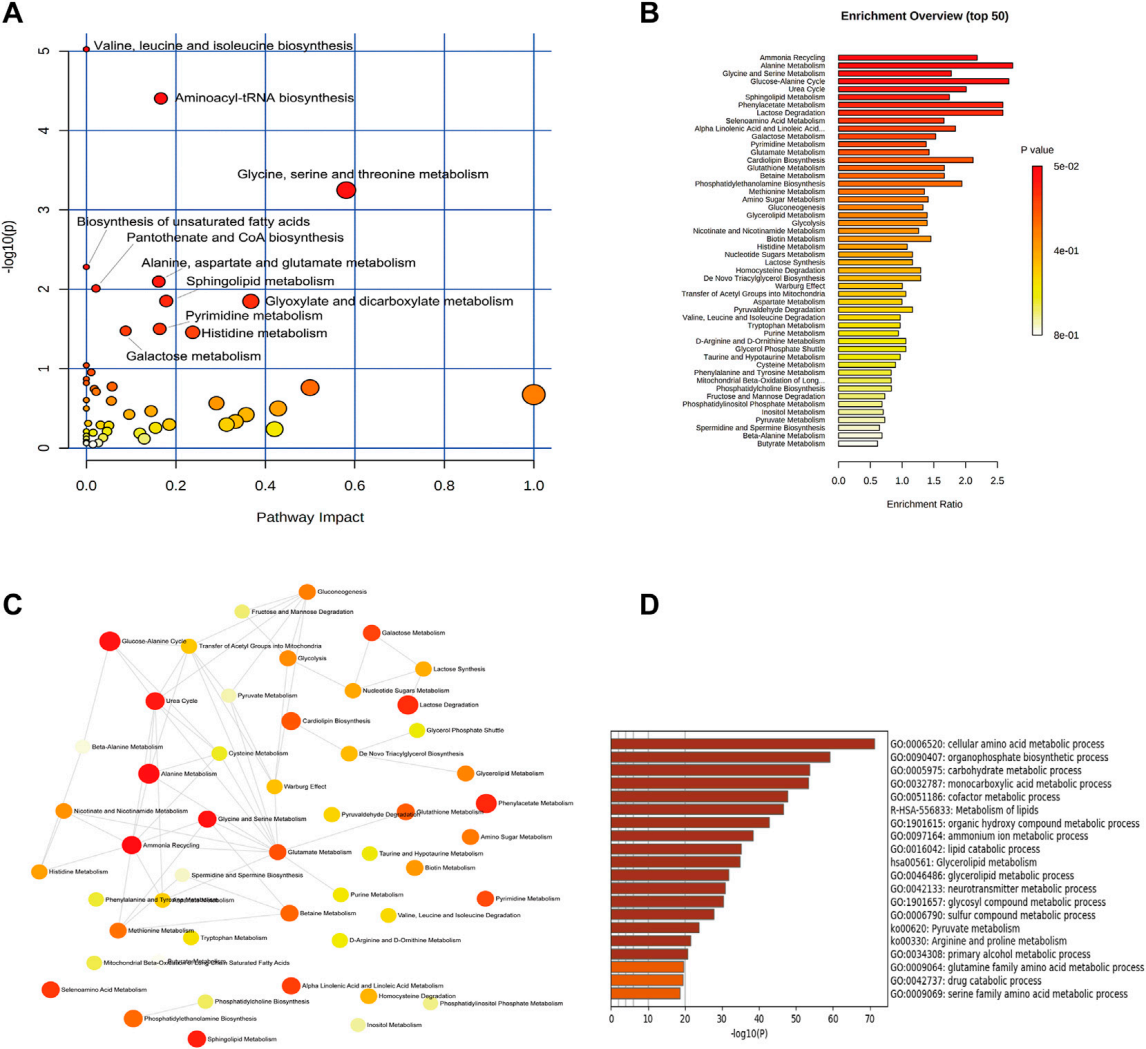
Enrichment analysis of metabolic pathway and related regulatory enzymes. **(A)** Bubble plots of altered metabolic pathways. **(B)** Overview of biological function related to the differential endogenous metabolites. **(C)** Network map of pathways. **(D)** Regulatory enzyme GO enrichment analysis results.

**FIGURE 5:**
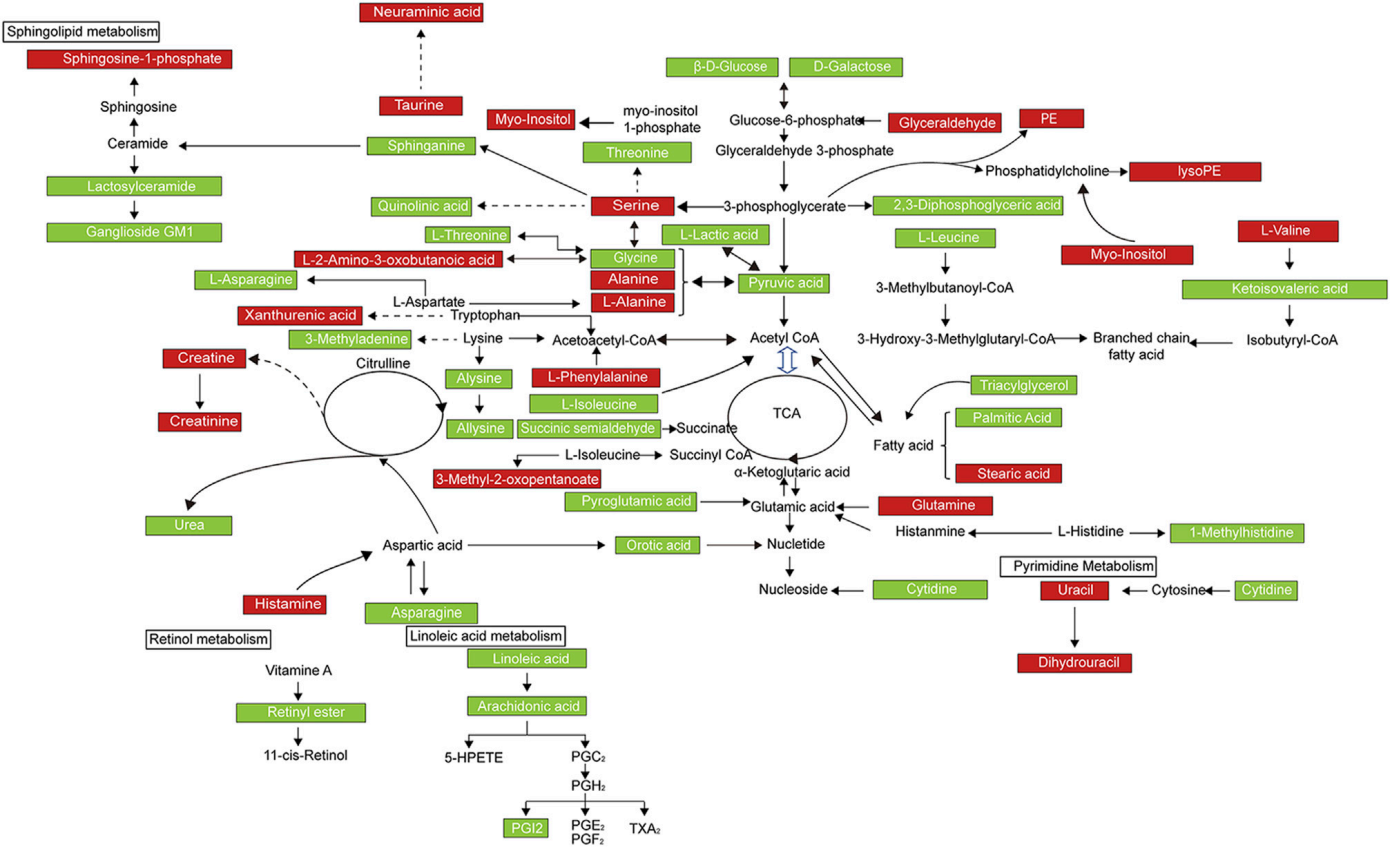
Metabolic network of the significantly changed endogenous metabolites in both serum and urine. Compared with the model group, elevated metabolites in the EDA group were represented by red, and the reduced metabolites were represented by green.

In order to comprehensively observe the changes in metabolic pathways, Metaboanalyst 4.0 (https://www.metaboanalyst.ca/) was applied for pathway and biological function enrichment by introducing all significant metabolites of serum and urine. The perturbed pathways including valine, leucine, and isoleucine biosynthesis, and phenylalanine, taurine, and hypotaurine metabolism were screened out (Figures 4A,B). And the correlations between biological functions are also shown in Figure 4C. The results suggested that EDA could improve many pathways under MCAO/R. Moreover, the interaction network of related regulatory enzymes built up with STRING (https://string-db.org/) is exhibited in Supplementary Figure S2A. And the GO enrichment analysis of related regulatory enzymes performed by Metascape (https://www.metascape.org/) showed that cellular amino acid metabolic process, monocarboxylic acid metabolic process, metabolism of lipids, and so on were regulated by EDA (Figure 4D), and the relations of them are exhibited in Supplementary Figure S2B. According to the results described previously, a schematic diagram of the changed metabolic pathways in serum and urine is exhibited in Figure 5.

### Semiquantitative Analysis of Taurine and Validation of CSAD Expression in Pre- and Posttreatment by EDA

**FIGURE 6:**
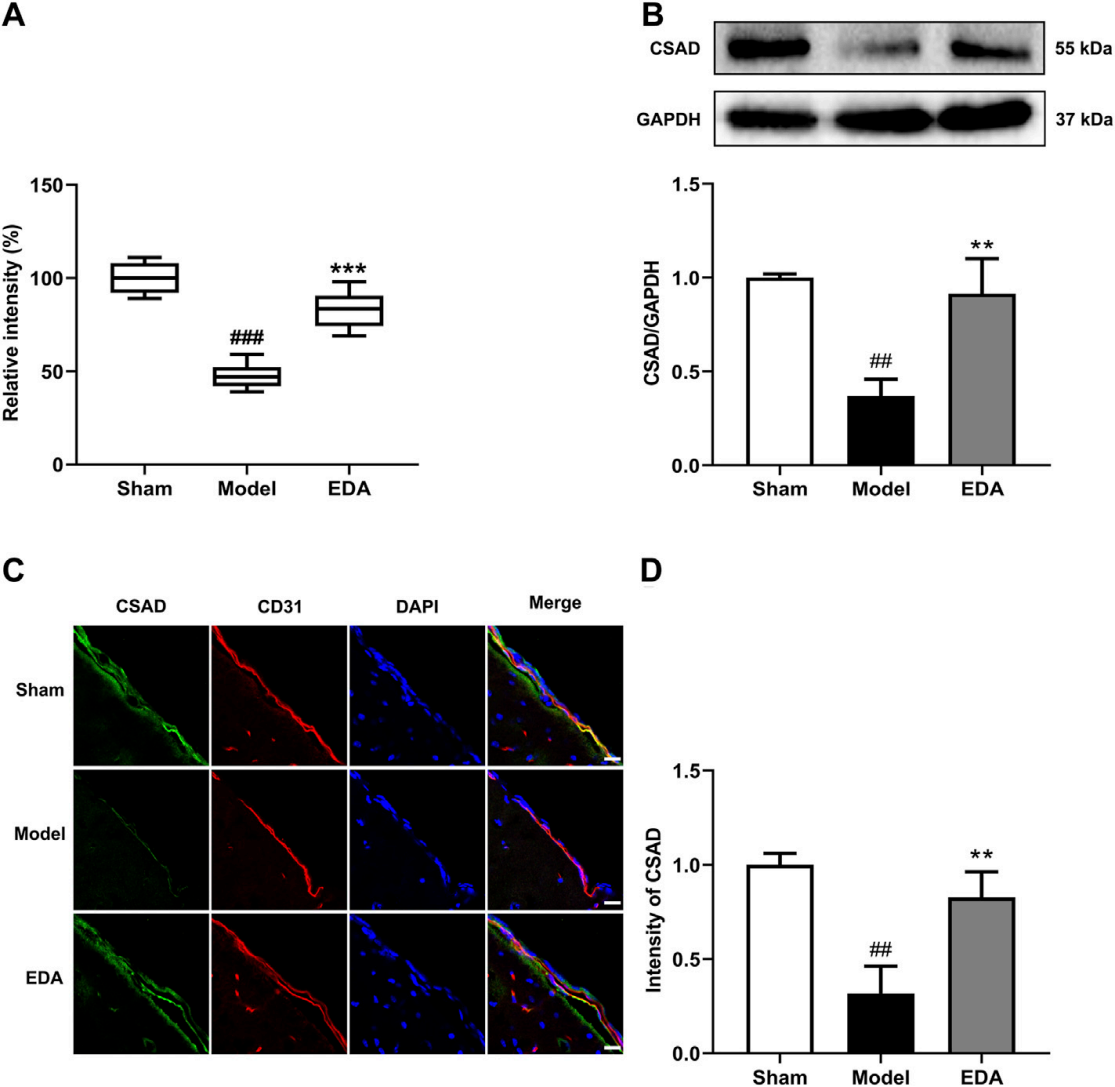
Effect of EDA on taurine and CSAD in MCAO/R mice. **(A)** Level of taurine in the different groups. **(B)** Representative Western blots and quantitative analyses of CSAD expression. **(C)** Immunofluorescence staining for CSAD (green) and CD31 (red) were performed on frozen brain sections, and the nuclei were counterstained with DAPI (blue) (scale bar = 20 μm). **(D)** Quantitative analyses of CSAD expression in endothelial cells. All data are presented as the means ± SEM, *n* = 6. ^#^ *p* < .05, ^##^ *p* < .01, ^###^ *p* < .001, *vs.* Sham group, *p < .05, **p < .01, ***p < .001, *vs.* MCAO/R group.

Identification of taurine was characterized by MS profile and confirmed with a standard compound, as shown in Supplementary Figure S3. Analyses of all samples showed that taurine decreased in MCAO/R mice compared with sham groups and could be improved by EDA (Figure 6A). To explore the possible reasons for the change of taurine, the level of CSAD, which is the predominant enzyme that regulates taurine biosynthesis in the brain, was determined. The expression of CSAD in the brain decreased in MCAO/R mice and dramatically increased in mice with the treatment of EDA (Figure 6B). The results of immunofluorescent staining proved the same tendency of CSAD expression in brain ECs (Figures 6C,D). These results demonstrate that the level of taurine was increased by EDA through promoting the expression of CSAD.

### EDA Alleviates MCAO/R Induced Brain EC Apoptosis *In Vivo*

**FIGURE 7:**
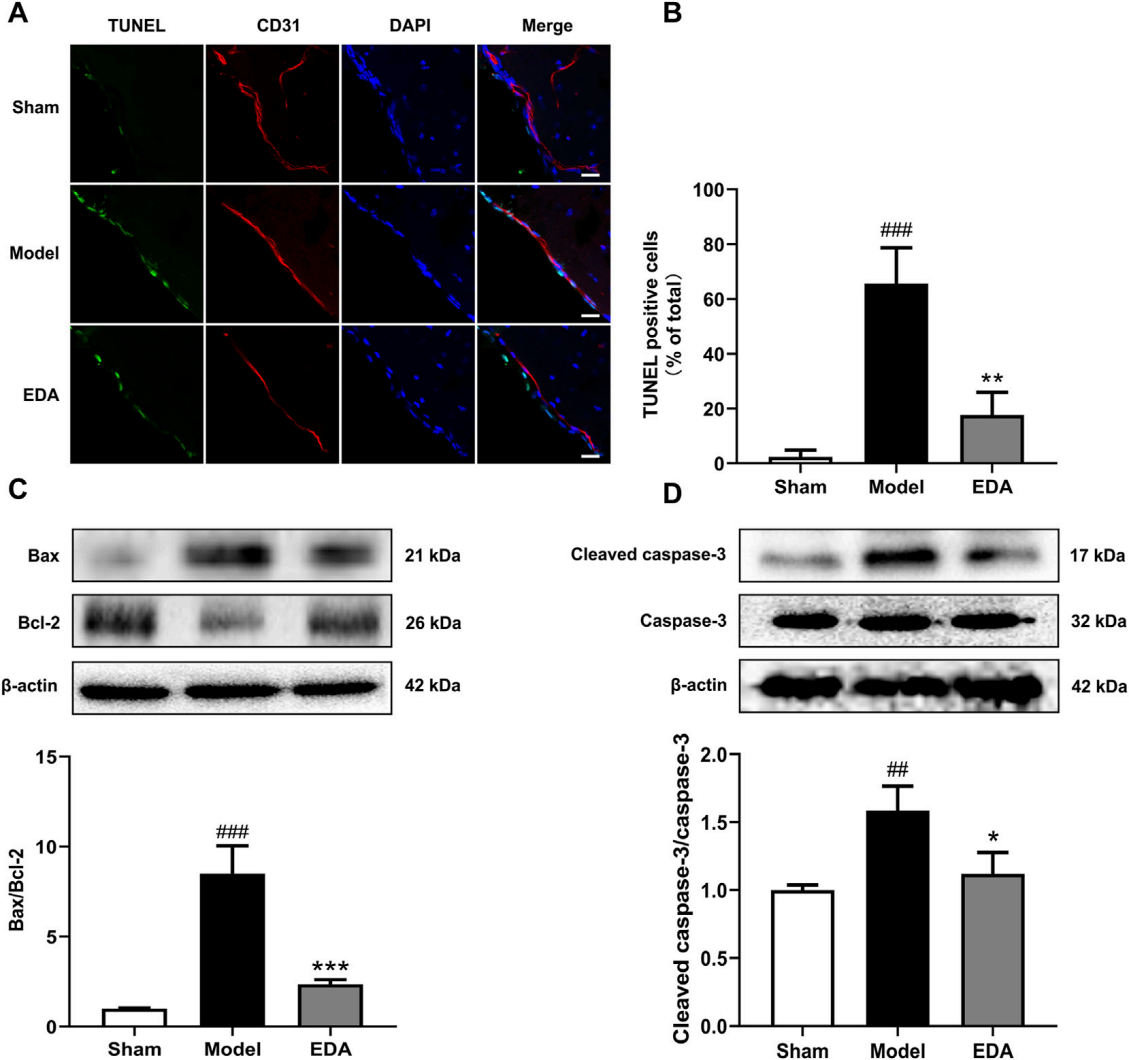
EDA mitigates cerebral endothelial apoptosis stimulated by MCAO/R in mice. **(A)** Brain-frozen sections were stained with TUNEL (green) and CD31 was used as a marker for endothelial cells; the nuclei were stained with DAPI (blue) (scale bar = 20 μm). **(B)** Quantitative analyses of apoptotic cells in endothelial cells. **(C)** Western blot analysis for the expression of Bax and Bcl-2 in brain tissues. **(D)** Western blot analysis for the expression of cleaved caspase-3 in brain tissues. All data are presented as the means ± SEM, *n* = 6. ^#^ *p* < .05, ^##^ *p* < .01, ^###^ *p* < .001, *vs.* Sham group, *p < .05, **p < .01, ***p < .001, *vs.* MCAO/R group.

As shown in Figures 7A,B, TUNEL assays of brain sections counterstained with CD31 to mark endothelium proved that TUNEL-positive brain ECs increased significantly in the MCAO/R mice, while the number of TUNEL-positive brain ECs was decreased after treatment with EDA. The levels of apoptosis proteins were measured with Western blot. The results showed that EDA significantly inhibited the expression of Bax and cleaved caspase-3, and upregulated the expression of Bcl-2 compared with the MCAO/R group (Figures 7C,D). These results suggested that EDA had a protective effect on MCAO/R-induced brain EC apoptosis.

## Discussion

In this study, through analysis of high-through metabolomics data and multistep validations, we attempted to find the untapped therapeutic targets of EDA, a first-line drug for the clinical treatment of stroke, toward elucidating the therapeutic mechanisms. Initially, we verified that EDA could significantly decrease cerebral infarction, inflammatory infiltration, neurological deficits, endothelium injury, and apoptosis in MCAO/R mice (Figure 1). The above investigations showed that EDA could effectively alleviate the cerebral ischemia-reperfusion injury in MCAO/R mice, thereby providing reliable samples for subsequent metabolomic analysis.

The results of metabolomic analyses presented the metabolic signature of EDA improvement of cerebral I/R injury, offering insights into the therapeutic mechanisms (Figure 5). The metabolites influenced by EDA were mainly lipids, fatty acids in serum, and were mainly amino acids in urine. Notably, oleic acid, linoleic acid, triacylglycerol (TG), palmitic acid, prostaglandin I2, urea, and leucine were reduced by EDA, while sphingosine-1-phosphate, taurine, valine, glutamine, and creatine were improved by EDA, especially taurine (Supplementary Table S1). The increased level of oleic acid leads to mitochondrial-derived reactive oxygen species production, resulting in endothelial dysfunction and blood–brain barrier disruption (Han et al., 2013; Gremmels et al., 2015). Linoleic acid associated with cardiovascular and cerebrovascular diseases significantly activates pro-inflammatory signaling in ECs, such as PI3K/Akt and ERK1/2, thus causing vessel inflammation, endothelial dysfunction, and death (Hennig et al., 2006; Bin et al., 2013; Satoh, 2013; Marchix et al., 2015). Adults with high triacylglycerol have increased risks of incident coronary heart disease and stroke, while lowering triglyceride levels of serum improves endothelial function, leading to a decrease in cardiovascular diseases (Hirano et al., 2008; Kajikawa et al., 2016; Lee et al., 2017). Similarly, the elevated palmitic acid level is related to the development of inflammation and endothelial dysfunction (Yang et al., 2019). Palmitic acid also induces energy metabolism disorders and apoptosis *via* activation of the apoptotic mitochondrial pathway (Adrian et al., 2017; Wen et al., 2017). Additionally, excess prostaglandin I2, urea, and leucine could similarly result in vascular endothelial injury and even lead to the disruption of barrier (Dorovini-Zis et al., 1987; De Bock et al., 2013; Lau and Vaziri, 2017; d’Apolito et al., 2018; Zhenyukh et al., 2018). Vessel inflammation, endothelial dysfunction, and death were the main factors causing cardiovascular and cerebrovascular diseases including stroke. Thus, reducing levels of differential metabolites damaging ECs is the potential mechanism of EDA for improving I/R injury. Sphingosine-1-phosphate, a bioactive intermediate of the sphingolipid metabolism, serves important physiological functions, such as proliferation, differentiation, survival, and migration, and is a key regulator of lymphocyte trafficking, endothelial barrier function, and vascular tone (Książek et al., 2015). Taurine, a semi-essential sulfur-containing amino acid, is present in several organs including the brain and has extensive physiological activities such as anti-inflammation and anti-oxidative stress, as well as regulation of energy metabolism, gene expression, osmosis, and quality control of protein. Thus, taurine protects against injuries of ECs and has potential ameliorating effects against cardiovascular diseases and neurological disorder events such as neurodegenerative diseases, stroke, and diabetic neuropathy (Ulrich-Merzenich et al., 2007; Murakami, 2014; Jakaria et al., 2019). Additionally, taurine has been reported to have a protective effect on the brain in stroke by down-regulating PARP and NF-κB, and activating GABAA and glycine receptors, as well as attenuating cell death (Wang GH. et al., 2007; Sun et al., 2012). Valine, one of the eight essential amino acids and sugar-producing amino acids for the human body, could promote the normal growth of the body, regulate protein and energy metabolism, and neurological functions (Shimomura and Kitaura, 2018). Glutamine metabolism is important for ECs in health and disease conditions, especially in cardiovascular diseases. Glutamine not only possesses potent antioxidant and anti-inflammatory effects in the circulation but also drives key processes in vascular cells, including proliferation, migration, apoptosis, senescence, and extracellular matrix deposition by serving as a substrate for the synthesis of DNA, ATP, proteins, and lipids (Rohlenova et al., 2018; Durante, 2019). Creatine exhibits ergogenic effects under a number of conditions including neurodegenerative diseases by maintaining cellular ATP stores. Moreover, creatine could improve ischemic stroke and other cerebrovascular diseases due to antioxidant activity, neurotransmitter-like behavior, and prevention of the opening of the mitochondrial permeability pore (Balestrino et al., 2016). The metabolites described above could replicate some of the previous research findings of the treatment of stroke. In this study, the level of oleic acid and palmitic acid decreased after treating with EDA, which appears to be in line with the treatment of gross saponins of *Tribulus terrestris* fruit (Wang et al., 2019). Similarly, the decrease of phenylalanine was in accordance with the previous report (Fu et al., 2019). Therefore, the possible mechanisms of EDA improvement of stroke were anti-inflammation and anti-oxidative stress, as well as a decrease of endothelial dysfunction and blood–brain barrier disruption by regulating the metabolites described above.

Subsequently, the pathways mediated by EDA were enriched with the differential metabolites. The results highlighted amino acid metabolisms, fatty acid metabolisms, and lipid metabolisms, such as valine, leucine, and isoleucine biosynthesis, biosynthesis of unsaturated fatty acids, sphingolipid metabolism as well as taurine and hypotaurine metabolism pathways (Figures 4A–C). As described above, valine, leucine, and isoleucine metabolism and most fatty acid metabolisms were directly associated with endothelial dysfunction through increasing reactive oxygen species generation and inflammation, and the change of these pathways, as well as taurine and hypotaurine metabolism, were the important pathological factors in stroke (Hennig et al., 2006; Gremmels et al., 2015; Zhenyukh et al., 2018). Consequently, EDA mainly improves endothelial dysfunction and blood–brain barrier function by interfering with these metabolic pathways, which might be the metabolism mechanism of EDA alleviating cerebral impairment induced by ischemia-reperfusion.

ECs are the key part of the blood–brain barrier which maintains the normal function of the central nervous system and metabolism activity of brain tissue. The death of ECs occurs at the primary stage of stroke which plays a vital role in the early impairment of neurological functions and may interfere with later recovery (Zille et al., 2019). Thus, EC is the potential target mechanism for the treatment of stroke. Interestingly, taurine, one of the mainly increased metabolites by EDA, possesses the effect of endothelium protection. EDA might inhibit the death of ECs by increasing the level of taurine. Hence, taurine was selected for the follow-up validation to explore the potential targets of EDA. Our research demonstrated that EDA could effectively elevate the level of taurine. In order to further confirm the mechanism of EDA increase of taurine, the key regulatory enzymes of taurine were verified. CSAD is the key synthetase of taurine and expresses in the brain, while its biofunction in MCAO/R has not been clarified yet (Park et al., 2014). We found that EDA could significantly increase the expression of CSAD, which indicated that EDA might elevate the level of taurine in MCAO/R mice by increasing CSAD. Therefore, CSAD is a potential target of EDA therapy for stroke.

Apoptosis is a common way of cell death, and the apoptosis of ECs is an important pathological process in stroke (Yang et al., 2018). Bax, Bcl-2, and cleaved caspase-3 are the characteristic proteins of apoptosis. We found that EDA could effectively inhibit Bax, Bcl-2, and cleaved caspase-3 by increasing taurine (Figure 7). Thus, EDA inhibited apoptosis of ECs and ameliorated cerebral microvascular endothelial dysfunction, thereby alleviating brain injury induced by I/R. Meanwhile, the EC apoptosis was inhibited along with the expression of CSAD increasing. In summary, taurine and CSAD have a critical role in inhibiting ECs apoptosis, which might be an important metabolism mechanism of EDA treatment stroke.

Our current study still has several limitations. EDA treats stroke with many complex mechanisms. Numerous differential metabolites and pathways were found to be associated with therapeutic stroke of EDA. Thus, more differential metabolites need to be further investigated.

## Conclusion

In the present study, a functional metabolomics strategy was used to characterize metabolite signatures and their underlying mechanisms associated with the therapeutic stroke of EDA. We not only constructed the differential metabolic network map providing clues for investigating mechanisms but also identified the biological function of taurine in the process of EDA improving stroke. It is interesting to note that taurine and its regulatory enzyme CSAD seem to play a key role in inhibiting EC apoptosis induced by I/R. Therefore, this study elucidated that EDA improves stroke *via* the influence of metabolites and provided a potential therapeutic target for stroke.

## Data Availability

The original contributions presented in the study are included in the article/Supplementary Material, further inquiries can be directed to the corresponding authors.

## Abbreviations

CSAD, cysteine sulfinic acid decarboxylase; EDA, edaravone; LDH, lactate dehydrogenase; MCAO/R, middle cerebral artery occlusion/reperfusion; TUNEL, TdT-mediated dUTP nick-end labeling; H&E, hematoxylin and eosin; EC, endothelial cell; I/R, ischemia/reperfusion.
