# Supplementary material for: Metabolomic Profiling of Brain Protective Effect of Edaravone on Cerebral Ischemia-Reperfusion Injury in Mice
Source: Front Pharmacol. 2022 Feb 14;13:814942. doi: 10.3389/fphar.2022.814942 (PMC8882761; doi:10.3389/fphar.2022.814942)
Supplement: Supplementary file 1 [file DataSheet1.docx]

**Metabolomic profiling of brain protective effect of edaravine on cerebral ischemia-reperfusion injury in mice**


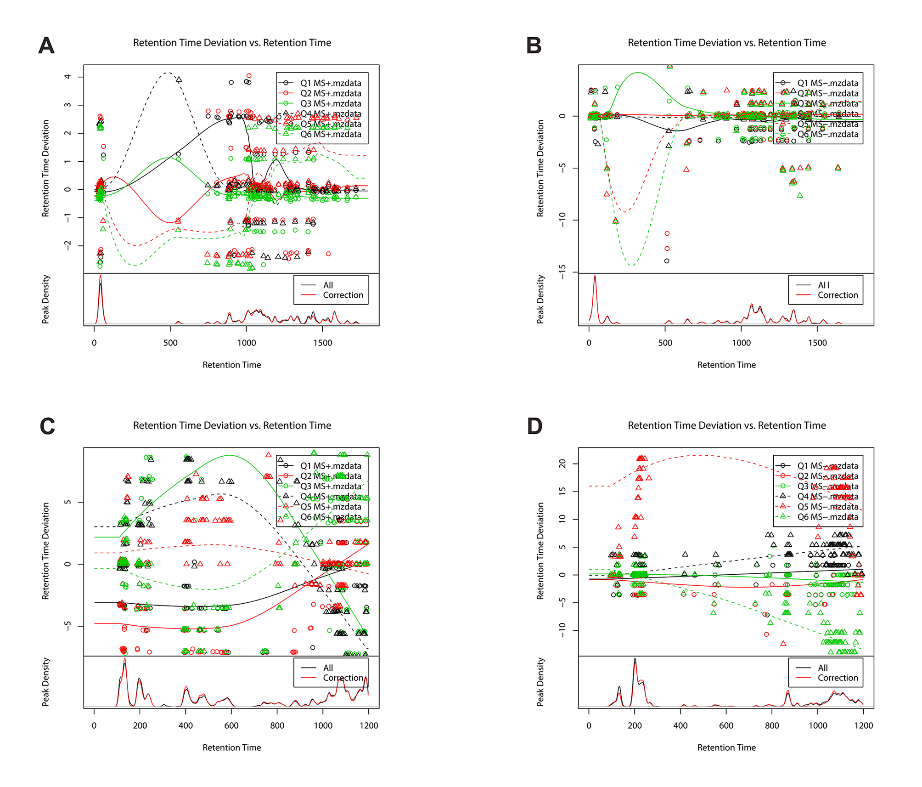


**Supplementary Fig. 1. Retention time deviation of the QC samples.**

**­­­**


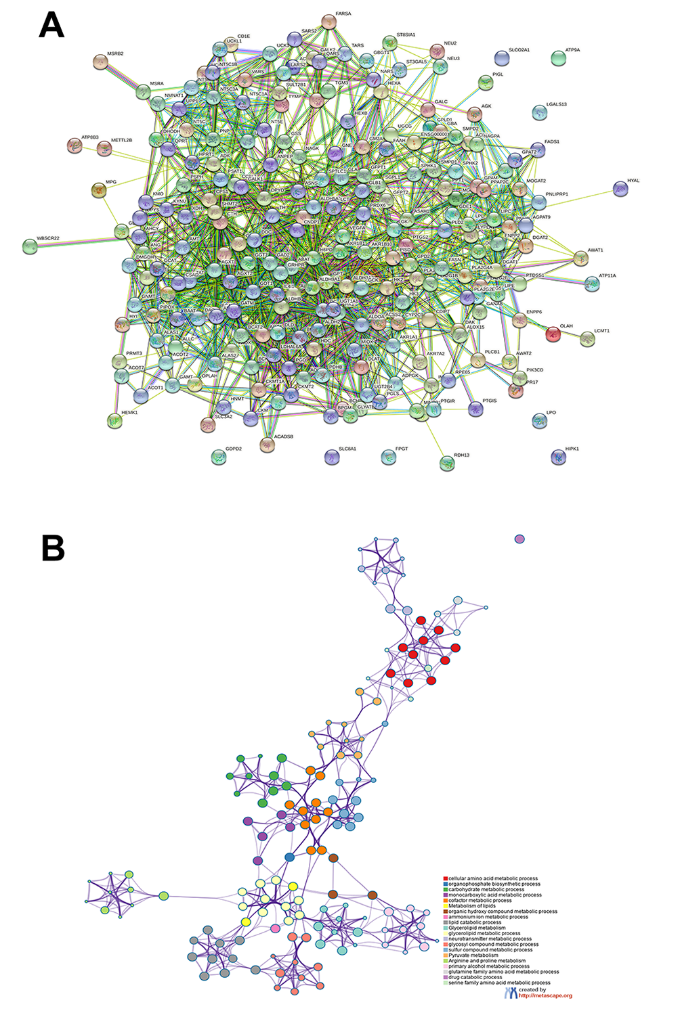


**Supplementary Fig. 2. Enrichment analysis of related regulatory enzymes.** (A) Regulatory protein network map. (B) The hierarchically clustered tree based on Kappa-statistical similarities among their gene memberships.


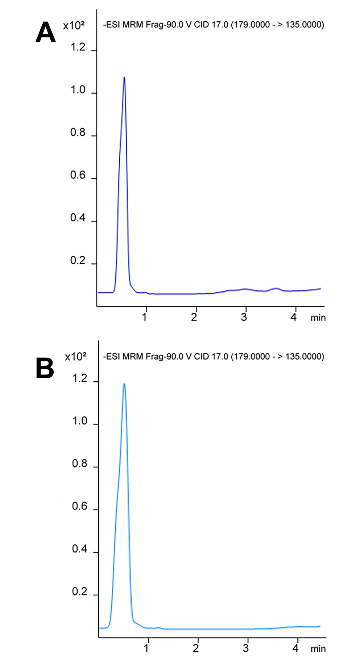


**Supplementary Fig. 3. Representative multiple reaction monitoring (MRM) chromatograms of taurine.** (A) The chromatogram of taurine obtained from urine samples. (B) The chromatogram of taurine obtained from standard solution.

**Supplementary Table 1.** Statistical analysis of 51 and 56 differential metabolites from the comparison of model versus EDA in serum and urine, respectively.

| **Ion mode** | **Identification** | **m/z** | **Rt(min)** | **VIP** | **P value** | **Fold change** | **Metabolic pathways** | **Enzymes** | **Genes** |
| --- | --- | --- | --- | --- | --- | --- | --- | --- | --- |
| Serum Positive | Uracil | 261.0004562 | 0.677217047 | 1.58831 | 0.0059542 | 1.2644 | Pyrimidine Metabolism；Beta-Alanine Metabolism | Purine nucleoside phosphorylase; Uridine phosphorylase 1; Uridine-cytidine kinase-like 1; Uracil nucleotide/cysteinyl leukotriene receptor | PNP; UPP1; UCKL1; GPR17 |
|  |  |  |  |  |  |  |  |  |  |
|  |  |  |  |  |  |  |  |  |  |
|  |  |  |  |  |  |  |  |  |  |
|  |  |  |  |  |  |  |  |  |  |
|  | Allysine | 175.1208642 | 0.677566316 | 1.26564 | 0.00050853 | 0.85229 | Lysine Degradation | Aldehyde oxidase; Alpha-aminoadipic semialdehyde dehydrogenase | AOX1; ALDH7A1; |
|  | L-Leucine | 234.0293333 | 0.719537652 | 1.50334 | 9.20E-05 | 0.75385 | Valine, Leucine and Isoleucine Degradation | Branched-chain-amino-acid aminotransferase, cytosolic; Leucine--tRNA ligase, cytoplasmic; 4F2 cell-surface antigen heavy chain; Leucine carboxyl methyltransferase 1 | BCAT1; LARS; SLC3A2 LCMT1 |
|  | Ketoisovaleric acid | 133.0970427 | 0.756734856 | 1.56415 | 0.0015708 | 0.75249 | Valine, Leucine and Isoleucine Degradation | 2-oxoisovalerate dehydrogenase subunit alpha, mitochondrial; Branched-chain-amino-acid aminotransferase, cytosolic | BCKDHA; BCAT1 |
|  | L-Phenylalanine | 86.09793862 | 0.76171122 | 1.5821 | 0.00099401 | 1.74673 | Phenylalanine and Tyrosine Metabolism | Tyrosine 3-monooxygenase; Phenylalanine--tRNA ligase alpha subunit; Tyrosine aminotransferase; Aromatic-L-amino-acid decarboxylase; Lactoperoxidase; Peroxiredoxin-6; Aspartate aminotransferase, cytoplasmic; Angiotensin-converting enzyme 2; | TH; FARSA; TAT; DDC; LPO; PRDX6; GOT1; ACE2 |
|  | L-Isoleucine | 127.0382261 | 0.761794639 | 1.62409 | 2.06E-06 | 0.62584 | Valine, leucine and isoleucine degradation；Valine, leucine and isoleucine biosynthesis | Branched-chain-amino-acid aminotransferase, cytosolic; Short/branched chain specific acyl-CoA dehydrogenase, mitochondrial | BCAT1; ACADSB |
|  | Prostaglandin I2 | 188.0728312 | 0.968168032 | 1.06975 | 0.00035431 | 0.83265 | Arachidonic Acid Metabolism | Prostaglandin G/H synthase 2; Prostacyclin synthase; Prostacyclin receptor | PTGS2; PTGIS; PTGIR |
|  | D-Glucose | 227.0818847 | 0.973614997 | 1.1822 | 0.0015149 | 0.84358 | Glycolysis;Gluconeogenesis | Glucokinase; Hexokinase-3; Hexokinase-2; Hexokinase-1; GDH/6PGL endoplasmic bifunctional protein; Neutral alpha-glucosidase AB; ADP-dependent glucokinase | GCK; HK3; HK2; HK1; H6PD; GANAB; ADPGK |
|  | PE(14:1(9Z)/14:1(9Z)) | 614.352802 | 12.3772456 | 1.63358 | 2.40E-16 | 0.017068 | Phosphatidylcholine Biosynthesis | Cytosolic phospholipase A2; Phospholipase D2 | PLA2G4A; PLD2 |
|  | Sphinganine | 247.4778074 | 14.65771804 | 1.33224 | 0.0086414 | 0.78797 | Sphingolipid Metabolism | Lipid phosphate phosphohydrolase 2; Globoside alpha-1,3-N-acetylgalactosaminyltransferase 1; Sphingosine kinase 1 | PPAP2C; GBGT1; SPHK1 |
|  | Urea | 380.2597817 | 16.96546046 | 1.02288 | 0.00075513 | 0.84493 | Arginine and Proline Metabolism | Arginase-1; Agmatinase, mitochondrial; Probable allantoicase; | ARG1; AGMAT; ALLC |
|  | LysoPE(20:0/0:0) | 1015.684943 | 18.06459834 | 1.07179 | 0.0031294 | 1.1357 | - | Ectonucleotide pyrophosphatase/phosphodiesterase family member 2 | ENPP2 |
|  | LysoPE(22:0/0:0) | 538.3933425 | 20.94742283 | 1.60775 | 0.0027001 | 1.2875 | - | Ectonucleotide pyrophosphatase/phosphodiesterase family member 2 | ENPP2 |
|  | Ganglioside GM1 (d18:1/14:0) | 353.2720997 | 21.56844446 | 1.27299 | 0.012694 | 1.6051 | - | Alpha-N-acetylneuraminide alpha-2,8-sialyltransferase; Sialidase-3; Globoside alpha-1,3-N-acetylgalactosaminyltransferase 1; N-acetylglucosaminyl-phosphatidylinositol de-N-acetylase; Lactosylceramide alpha-2,3-sialyltransferase | ST8SIA1; NEU3; GBGT1; PIGL; ST3GAL5 |
|  | PE(24:1(15Z)/15:0) | 552.4092245 | 22.27088094 | 1.61646 | 2.19E-05 | 1.5806 | Phosphatidylcholine Biosynthesis | Calcium-dependent phospholipase A2; Cytosolic phospholipase A2; Phospholipase D2 | PLA2G5; PLA2G4A; PLD2 |
|  | PE(15:0/24:1(15Z)) | 553.412773 | 22.28708902 | 1.61904 | 1.27E-05 | 1.6148 | Phosphatidylcholine Biosynthesis | Calcium-dependent phospholipase A2; Phospholipase D2 | PLA2G5; PLD2 |
|  | 2,3-Diphosphoglyceric acid | 829.563655 | 22.3549248 | 1.62913 | 1.72E-12 | 0.019641 | Glycolysis；Gluconeogenesis | Bisphosphoglycerate mutase; Phosphoglycerate mutase 2; Multiple inositol polyphosphate phosphatase 1 | BPGM; PGAM2; MINPP1 |
|  | PE(22:1(13Z)/15:0) | 240.242737 | 23.49589127 | 1.58771 | 0.0027867 | 1.2398 | Phosphatidylcholine Biosynthesis | Calcium-dependent phospholipase A2; Phospholipase D2 | PLA2G5; PLD2 |
|  | PE(24:0/15:0) | 109.1020784 | 23.50443541 | 1.54005 | 0.0020513 | 1.2654 | Phosphatidylcholine Biosynthesis | Calcium-dependent phospholipase A2; Phospholipase D2 | PLA2G5; PLD2 |
|  | Oleic Acid | 284.2813766 | 24.00496166 | 1.3654 | 0.003065 | 1.2222 | - | Fatty acid synthase; Bile acid-CoA:amino acid N-acyltransferase; Cytosolic acyl coenzyme A thioester hydrolase; Fatty-acid amide hydrolase 1; 2-acylglycerol O-acyltransferase 2 | FASN; BAAT; ACOT7; FAAH; MOGAT2 |
|  | MG(20:3(8Z,11Z,14Z)) | 341.3089893 | 24.02029616 | 1.49279 | 0.00077579 | 1.7084 | - | Monoglyceride lipase; Hormone-sensitive lipase; 2-acylglycerol O-acyltransferase 2; Acylglycerol kinase, mitochondrial; Ectonucleotide pyrophosphatase/phosphodiesterase family member 6 | MGLL; LIPE; MOGAT2; AGK; ENPP6 |
|  | DG(22:5(7Z,10Z,13Z,16Z,19Z)/22:4(7Z,10Z,13Z,16Z)) | 267.271272 | 25.53206336 | 1.40825 | 0.0093224 | 1.4491 | Phosphatidylcholine Biosynthesis | Diacylglycerol kinase theta; 1-phosphatidylinositol 4,5-bisphosphate phosphodiesterase beta-1; Hepatic triacylglycerol lipase; Inactive pancreatic lipase-related protein 1 | DGKQ; PLCB1; LIPC; PNLIPRP1 |
|  | N1,N12-Diacetylspermine | 285.2829773 | 25.53917891 | 1.45754 | 0.0056474 | 1.4501 | - | Peroxisomal N(1)-acetyl-spermine/spermidine oxidase | PAOX |
| Serum Negative | D-Galactose | 104.9594042 | 0.551507986 | 1.0994 | 0.00080482 | 0.88552 | Galactose Metabolism；Nucleotide Sugars Metabolism | Aldose reductase; Hexokinase-3; Hexokinase-2; Hexokinase-1; N-acetylgalactosamine kinase; Galactokinase; Lactase-phlorizin hydrolase | AKR1B1; HK3; HK2; HK1; GALK2; GALK1; LCT |
|  | L-Lactic acid | 102.9980328 | 0.634827889 | 1.43324 | 6.99E-06 | 0.69699 | Gluconeogenesis；Pyruvate Metabolism | L-lactate dehydrogenase A-like 6A; L-lactate dehydrogenase B chain | LDHAL6A; LDHB |
|  | Glyceraldehyde | 216.0367796 | 0.677639968 | 1.41659 | 0.00085279 | 1.2121 | Glycerolipid Metabolism；Fructose and Mannose Degradation | Aldose reductase; 4-trimethylaminobutyraldehyde dehydrogenase; Alpha-aminoadipic semialdehyde dehydrogenase; Aldehyde dehydrogenase, mitochondrial; Fatty aldehyde dehydrogenase; Alcohol dehydrogenase [NADP(+)]; Fructose-bisphosphate aldolase A; Bifunctional ATP-dependent dihydroxyacetone kinase/FAD-AMP lyase (cyclizing) | AKR1B1; ALDH9A1; ALDH7A1; ALDH2; ALDH3A2; AKR1A1; ALDOA; DAK |
|  | Pyruvic acid | 87.00936944 | 0.720009537 | 1.01495 | 0.0064389 | 0.78503 | Urea Cycle；Glycine and Serine Metabolism；Pyruvate Metabolism | Pyruvate dehydrogenase E1 component subunit beta, mitochondrial; Pyruvate carboxylase, mitochondrial;  Dihydrolipoyl dehydrogenase, mitochondrial; Dihydrolipoyllysine-residue acetyltransferase component of pyruvate dehydrogenase complex, mitochondrial; D-amino-acid oxidase; 4-aminobutyrate aminotransferase, mitochondrial Alanine--glyoxylate aminotransferase 2, mitochondrial | PDHB; PC; DLD; DLAT; DAO; ABAT; AGXT2 |
|  | Orotic acid | 115.0380547 | 1.012413599 | 1.10343 | 0.0022569 | 0.82601 | Pyrimidine Metabolism； | Dihydroorotate dehydrogenase (quinone), mitochondrial; Uridine 5'-monophosphate synthase | DHODH; UMPS |
|  | 2-Hydroxy-3-methylbutyric acid | 117.0527059 | 1.017097415 | 1.02935 | 0.0046193 | 0.84871 | valine, leucine, and isoleucine metabolism | Sulfotransferase family cytosolic 2B member 1; UDP-glucuronosyltransferase 1-1 | SULT2B1; UGT1A1 |
|  | 3-Methyl-2-oxovaleric acid | 129.0555807 | 1.73255806 | 1.42944 | 5.1375E-06 | 0.64463 | Valine, Leucine and Isoleucine Degradation | 2-oxoisovalerate dehydrogenase subunit beta, mitochondrial; Branched-chain-amino-acid aminotransferase, cytosolic; L-amino-acid oxidase | BCKDHB; BCAT1; IL4I1 |
|  | PIP(20:1(11Z)/16:0) | 131.0711456 | 1.94090544 | 1.43794 | 1.48E-06 | 0.69312 | - | 1-phosphatidylinositol 4,5-bisphosphate phosphodiesterase beta-1; N-acetylglucosaminyl-phosphatidylinositol de-N-acetylase; Probable phospholipid-transporting ATPase IH; Phosphatidylinositol-4,5-bisphosphate 3-kinase catalytic subunit delta isoform ; Phosphatidylserine synthase 1; Phosphatidylserine decarboxylase proenzyme | PLCB1; PIGL; ATP11A; PIK3CD; PTDSS1; PISD |
|  | TG(12:0/12:0/12:0) | 201.1125621 | 12.50969798 | 1.49382 | 7.59E-06 | 0.7431 | Triacylglycerol Biosynthesis | Hepatic triacylglycerol lipase; Pancreatic triacylglycerol lipase; Endothelial lipase; Diacylglycerol O-acyltransferase 1; Glycerol-3-phosphate acyltransferase 3 | LIPC; PNLIP; LIPG; DGAT1; AGPAT9 |
|  | Sphingosine-1-phosphate | 378.2404994 | 17.02466243 | 1.42888 | 3.65E-07 | 1.82795 | Sphingolipid Metabolism | Lipid phosphate phosphohydrolase 2; Globoside alpha-1,3-N-acetylgalactosaminyltransferase 1; Sphingosine-1-phosphate lyase 2; Sphingosine kinase 1; Ganglioside GM2 activator | PPAP2C; GBGT1; SGPL1; SPHK2; GM2A |
|  | LysoPE(0:0/16:0) | 512.2974515 | 17.15223447 | 1.23659 | 0.007252 | 1.1411 | - | Ectonucleotide pyrophosphatase/phosphodiesterase family member 2 | ENPP2 |
|  | LysoPE(22:6(4Z,7Z,10Z,13Z,16Z,19Z)/0:0) | 525.2798446 | 17.65737928 | 1.00925 | 0.0094118 | 0.76922 | - | Ectonucleotide pyrophosphatase/phosphodiesterase family member 2 | ENPP2 |
|  | Linoleic acid | 241.1801415 | 17.6997207 | 1.46586 | 1.90E-06 | 0.75034 | Alpha Linolenic Acid and Linoleic Acid Metabolism | Cytosolic phospholipase A2; Acyl-coenzyme A thioesterase 1; Arachidonate 15-lipoxygenase | PLA2G4A; ACOT1; ALOX15 |
|  | Cytidine | 588.3289399 | 17.86771342 | 1.4495 | 0.00032095 | 0.90288 | Pyrimidine Metabolism | 5'-nucleotidase; 5'(3')-deoxyribonucleotidase, cytosolic type; 5'(3')-deoxyribonucleotidase, mitochondrial; Bifunctional UDP-N-acetylglucosamine 2-epimerase/N-acetylmannosamine kinase; Uridine-cytidine kinase 2; Cytosolic 5'-nucleotidase 3 | NT5E; NT5C; NT5M; GNE; UCK2; NT5C3 |
|  | LysoPE(20:2(11Z,14Z)/0:0) | 613.3315375 | 17.90968894 | 1.11913 | 0.0037389 | 0.8646 | - | Ectonucleotide pyrophosphatase/phosphodiesterase family member 2 | ENPP2 |
|  | LysoPE(24:6(6Z,9Z,12Z,15Z,18Z,21Z)/0:0) | 612.328392 | 17.90969151 | 1.09942 | 0.0046536 | 0.86618 | - | Ectonucleotide pyrophosphatase/phosphodiesterase family member 2 | ENPP2 |
|  | Palmitic Acid | 812.5416261 | 18.20379887 | 1.33251 | 0.00010228 | 0.84063 | Glycerolipid Metabolism；Fatty acid Metabolism；Fatty Acid Biosynthesis | Fatty acid synthase; Acyl-protein thioesterase 1; Galactoside-binding soluble lectin 13; Hepatic triacylglycerol lipase; Lipoprotein lipase; Acyl-coenzyme A thioesterase 2, mitochondrial; Palmitoyl-protein thioesterase 1; Alkaline ceramidase 1 | FASN; LYPLA1; LGALS13; LIPC; LPL; ACOT2; PPT1; ACER1 |
|  | PE(20:3(8Z,11Z,14Z)/20:1(11Z)) | 840.573059 | 18.79485763 | 1.41596 | 0.00035704 | 1.1289 | Phosphatidylethanolamine Biosynthesis | Calcium-dependent phospholipase A2; Cytosolic phospholipase A2; Phospholipase D2;Probable phospholipid-transporting ATPase IIA | PLA2G5; PLA2G4A; PLD2; ATP9A |
|  | LysoPE(22:2(13Z,16Z)/0:0) | 552.3655925 | 19.17308057 | 1.26848 | 0.0080864 | 1.156 | - | Ectonucleotide pyrophosphatase/phosphodiesterase family member 2 | ENPP2 |
|  | LysoPE(0:0/18:0) | 480.3087361 | 19.21519854 | 1.10267 | 0.0024747 | 0.81519 | - | Ectonucleotide pyrophosphatase/phosphodiesterase family member 2 | ENPP2 |
|  | PE(22:2(13Z,16Z)/15:0) | 582.3762416 | 21.00821807 | 1.18619 | 0.011286 | 1.2124 | Phosphatidylethanolamine Biosynthesis | Calcium-dependent phospholipase A2; Cytosolic phospholipase A2; Phospholipase D2 | PLA2G5; PLA2G4A; PLD2 |
|  | Retinyl ester | 301.2166798 | 21.11288113 | 1.46855 | 1.38E-07 | 0.59522 | Retinol Metabolism | Diacylglycerol O-acyltransferase 1; Retinoid isomerohydrolase; Lecithin retinol acyltransferase; Diacylglycerol O-acyltransferase 2; Acyl-CoA wax alcohol acyltransferase 1; Acyl-CoA wax alcohol acyltransferase 2 | DGAT1; RPE65; LRAT; DGAT2; AWAT1; AWAT2 |
|  | alpha-Linolenic acid | 277.2169115 | 21.19775029 | 1.03454 | 0.0029427 | 0.84777 | Alpha Linolenic Acid and Linoleic Acid Metabolism | Calcium-dependent phospholipase A2; Phospholipase A2; Phospholipase A2, membrane associated; Acyl-coenzyme A thioesterase 2, mitochondrial; Transient receptor potential cation channel subfamily V member 1; Fatty acid desaturase 1; Sodium/calcium exchanger 1 | PLA2G5; PLA2G1B; PLA2G2A; ACOT2; TRPV1; FADS1; SLC8A1 |
|  | cis-9-Palmitoleic acid | 253.2167287 | 21.70312614 | 1.23144 | 7.87E-05 | 0.87546 | - | S-acyl fatty acid synthase thioesterase, medium chain | OLAH |
|  | Arachidonic Acid | 303.2326065 | 22.33542684 | 1.51275 | 3.88E-09 | 0.69515 | Alpha Linolenic Acid and Linoleic Acid Metabolism；Arachidonic Acid Metabolism | Fatty acid synthase; Calcium-dependent phospholipase A2; Group IIE secretory phospholipase A2; Cholinesterase; Prostaglandin G/H synthase 2; Arachidonate 15-lipoxygenase; Cytochrome P450 2C9; Acyl-coenzyme A thioesterase 2, mitochondrial; Homeodomain-interacting protein kinase 1; Solute carrier organic anion transporter family member 2A1 | FASN; PLA2G5; PLA2G2E; BCHE; PTGS2; ALOX15; CYP2C9; ACOT2; HIPK1; SLCO2A1 |
|  | PE(20:4(5Z,8Z,11Z,14Z)/20:2(11Z,14Z)) | 748.567445 | 24.50472328 | 1.48959 | 3.86E-08 | 0.53854 | Phosphatidylethanolamine Biosynthesis | Calcium-dependent phospholipase A2; Cytosolic phospholipase A2; Phospholipase D2 | PLA2G5; PLA2G4A; PLD2 |
|  | Lactosylceramide (d18:1/12:0) | 747.5639187 | 24.69437669 | 1.45742 | 5.61E-07 | 0.60327 | Sphingolipid Metabolism | Galactocerebrosidase; Sialidase-2; Lactase-phlorizin hydrolase; Globoside alpha-1,3-N-acetylgalactosaminyltransferase 1; N-acetylglucosaminyl-phosphatidylinositol de-N-acetylase; Beta-hexosaminidase subunit alpha | GALC; NEU2 LCT; GBGT1; PIGL |
|  | Stearic acid | 283.2639889 | 25.62080886 | 1.10383 | 0.020879 | 1.252 | Plasmalogen Synthesis | Fatty acid synthase; Phospholipase A2; Galactoside-binding soluble lectin 13; Lipoprotein lipase; Cholinesterase | FASN; PLA2G1B; LGALS13; LPL; BCHE |
| Urine  Positive | Glycine | 266.1392629 | 1.91191299 | 1.3193 | 0.0050714 | 0.75747 | Phenylalanine metabolism | Glycine N-acyltransferase | GLYAT |
|  | L-2-Amino-3-oxobutanoic acid | 220.1250042 | 2.043867132 | 1.19472 | 3.17E-06 | 1.6479 | Bile Acid Biosynthesis；Glycine and Serine Metabolism | 5-aminolevulinate synthase, erythroid-specific, mitochondrial; Glycine N-methyltransferase; Glycine amidinotransferase, mitochondrial; Glutathione synthetase | ALAS2; GNMT; GATM; GSS |
|  | N1-Methyl-4-pyridone-3-carboxamide | 366.2119717 | 3.327724914 | 1.22389 | 3.65E-07 | 2.2449 | Glycine and Serine Metabolism | 2-amino-3-ketobutyrate coenzyme A ligase, mitochondrial | GCAT |
|  | Xanthurenic acid | 217.1183335 | 3.78491058 | 1.13144 | 1.19E-05 | 1.8905 | Nicotinate and Nicotinamide Metabolism | Aldehyde oxidase | AOX1 |
|  | Dihydrouracil | 231.1341382 | 3.842643498 | 1.0718 | 1.86E-05 | 1.5713 | Tryptophan Metabolism | HemK methyltransferase family member 1 | HEMK1 |
|  | L-Alanine | 295.080338 | 3.854448546 | 1.32817 | 5.23E-09 | 1.6146 | Pyrimidine Metabolism；Beta-Alanine Metabolism | Dihydropyrimidinase; Dihydropyrimidine dehydrogenase [NADP(+)] | DPYS;DPYD |
|  | L-Asparagine | 133.0606544 | 3.944125984 | 1.30614 | 0.00019771 | 0.76346 | Urea Cycle；Alanine Metabolism | 4-aminobutyrate aminotransferase, mitochondrial; Kynureninase; Alanine--glyoxylate aminotransferase 2, mitochondrial; Serine--pyruvate aminotransferase; Alanine aminotransferase 1 | ABAT; KYNU; AGXT2; AGXT; GPT |
|  | Urea | 265.1144644 | 3.973855804 | 1.21986 | 5.11E-08 | 0.7504 | Aspartate Metabolism | Asparagine synthetase [glutamine-hydrolyzing]; Asparagine--tRNA ligase, cytoplasmic | ASNS; NARS |
|  | Creatinine | 184.1327791 | 3.99835747 | 1.27452 | 1.68E-08 | 1.903 | Arginine and Proline Metabolism；Urea Cycle | Arginase-1; Arginase-2, mitochondrial; Agmatinase, mitochondrial | ARG1; ARG2; AGMAT |
|  | L-Leucine | 130.1223184 | 6.46006754 | 1.28541 | 7.89E-05 | 0.28575 | Arginine and proline metabolism | - | - |
|  | Creatine | 132.0767663 | 7.642197202 | 1.25076 | 0.013933 | 2.62651 | Valine, Leucine and Isoleucine Degradation | Branched-chain-amino-acid aminotransferase, cytosolic; Branched-chain-amino-acid aminotransferase, mitochondrial; Probable leucine--tRNA ligase, mitochondrial | BCAT1; BCAT2; LARS2 |
|  | L-Valine | 249.1560119 | 9.411130796 | 1.38315 | 6.91E-07 | 22.648 | Glycine and Serine Metabolism；Arginine and Proline Metabolism | Guanidinoacetate N-methyltransferase; Creatine kinase S-type, mitochondrial; Creatine kinase B-type; Creatine kinase U-type, mitochondrial | GAMT; CKMT2; CKB; CKMT1A |
|  | 5-Methylcytidine | 411.1748865 | 9.541723768 | 1.25178 | 0.00017501 | 95.85 | Valine, Leucine and Isoleucine Degradation | Branched-chain-amino-acid aminotransferase, cytosolic; Branched-chain-amino-acid aminotransferase, mitochondrial; Valine--tRNA ligase | BCAT1; BCAT2; VARS |
|  | Neuraminic acid | 268.1044903 | 13.44437574 | 1.04702 | 0.0002053 | 8.3861 | Taurine and Hypotaurine Metabolism；Bile Acid Biosynthesis | Gamma-glutamyltranspeptidase 7 | GGT7 |
|  | Succinic semialdehyde | 398.0349867 | 16.57178699 | 1.37052 | 2.72E-11 | 0.22126 | - | UDP-glucuronosyltransferase 2B4 | UGT2B4 |
|  | Dimethylglycine | 273.0190971 | 17.23372203 | 1.36338 | 1.61E-10 | 2.6178 | Glutamate Metabolism | 4-aminobutyrate aminotransferase, mitochondrial; Succinate-semialdehyde dehydrogenase, mitochondrial; Aflatoxin B1 aldehyde reductase member 2 | ABAT; ALDH5A1; AKR7A2 |
|  | Myo-Inositol | 144.1017928 | 17.30273482 | 1.34963 | 9.08E-10 | 2.6362 | Glycine and Serine Metabolism；Methionine Metabolism | Dimethylglycine dehydrogenase, mitochondrial; Betaine--homocysteine S-methyltransferase 1 | DMGDH; BHMT |
|  | Pipecolic acid | 130.0861817 | 17.85690646 | 1.30818 | 0.0084592 | 0.76517 | Inositol Metabolism；Galactose Metabolism | Alpha-galactosidase A; CDP-diacylglycerol--inositol 3-phosphatidyltransferase; Inositol oxygenase; Inositol monophosphatase 1 | GLA; CDIPT; MIOX; IMPA1 |
|  | 3-Methyladenine | 231.1708839 | 17.95403109 | 1.33609 | 0.001685 | 0.73755 | lysine Metabolism | Peroxisomal sarcosine oxidase | PIPOX |
|  | 1-Methylhistidine | 110.029418 | 18.30439638 | 1.36341 | 0.00091045 | 0.78684 | - | DNA-3-methyladenine glycosylase | MPG |
|  | N-Acetyl-L-alanine | 302.0546438 | 18.54974959 | 1.09751 | 3.74E-06 | 1.7171 | Histidine Metabolism | Beta-Ala-His dipeptidase; Protein arginine N-methyltransferase 3 | CNDP1; PRMT3 |
|  | Pyroglutamic acid | 348.1256007 | 18.64825145 | 1.36538 | 5.95E-08 | 0.20397 | - | Carbamoyl-phosphate synthase [ammonia], mitochondrial; N-acetylglutamate synthase, mitochondrial | CPS1;NAGS |
|  | 6-(alpha-D-Glucosaminyl)-1D-myo-inositol | 342.1407686 | 19.04084183 | 1.35943 | 5.64E-08 | 0.24471 | Glutathione Metabolism | 5-oxoprolinase; Angiogenin; Vascular endothelial growth factor A | OPLAH; ANG; VEGFA |
|  | L-Glutamine | 148.0966116 | 19.56328833 | 1.32007 | 1.29E-09 | 3.5946 | - | Phosphatidylinositol-glycan-specific phospholipase D | GPLD1 |
|  | Hexanoylglycine | 245.0172192 | 19.72287272 | 1.31987 | 0.00047323 | 0.72467 | Pyrimidine Metabolism；Glutamate Metabolism；Purine Metabolism；Urea Cycle | Glutamine--fructose-6-phosphate aminotransferase [isomerizing] 2; Glutamine--fructose-6-phosphate aminotransferase [isomerizing] 1; Protein-glutamine gamma-glutamyltransferase E; Glutamine synthetase | GFPT2; GFPT1; TGM3; GLUL |
| Urine  Negative | Taurine | 124.9901562 | 14.84895046 | 1.27732 | 2.1282E-14 | **30.795** | Taurine and Hypotaurine Metabolism；Bile Acid Biosynthesis | Cysteine sulfinic acid decarboxylase | CSAD |
|  | Thiocysteine | 212.0039946 | 2.209298187 | 1.07207 | 0.026042 | 1.1497 | - | Glycine N-acyltransferase | GLYAT |
|  | 3-Methyl-2-oxovaleric acid | 172.1002034 | 2.264506571 | 1.07456 | 0.027988 | 1.1072 | - | Cystathionine gamma-lyase | CTH |
|  | β-Hydroxypyruvic acid | 103.0021918 | 2.295425591 | 1.0267 | 6.59E-10 | 1.8059 | Valine, Leucine and Isoleucine Degradation | 2-oxoisovalerate dehydrogenase subunit beta, mitochondrial; Branched-chain-amino-acid aminotransferase, cytosolic; L-amino-acid oxidase | BCKDHB; BCAT1; IL4I1 |
|  | 6-Phosphogluconic acid | 512.9661833 | 3.28426779 | 1.1501 | 2.27E-07 | 2.3212 | Glycine and Serine Metabolism | Glyoxylate reductase/hydroxypyruvate reductase; Serine--pyruvate aminotransferase; Putative hydroxypyruvate isomerase | GRHPR AGXT HYI |
|  | Galactitol | 652.9211557 | 3.33006014 | 1.08814 | 0.0051869 | 0.4186 | Pentose Phosphate Pathway | 6-phosphogluconolactonase; GDH/6PGL endoplasmic bifunctional protein; 6-phosphogluconate dehydrogenase, decarboxylating | PGLS; H6PD; PGD |
|  | Quinolinic acid | 228.0096886 | 3.41146551 | 1.27161 | 5.11E-11 | 0.084773 | Galactose Metabolism | Aldose reductase; Aldo-keto reductase family 1 member B10 | AKR1B1; AKR1B10 |
|  | 5-Hydroxyindoleacetaldehyde | 348.1776735 | 3.553553551 | 1.23429 | 1.31E-05 | 0.53048 | Nicotinate and Nicotinamide Metabolism；Tryptophan Metabolism | Kynurenine 3-monooxygenase | KMO |
|  | Cytidine | 129.0322652 | 3.617307245 | 1.19003 | 0.0023134 | 0.77035 | Tryptophan Metabolism | Aldehyde oxidase; 4-trimethylaminobutyraldehyde dehydrogenase; Alpha-aminoadipic semialdehyde dehydrogenase; Aldehyde dehydrogenase, mitochondrial | AOX1; ALDH9A1; ALDH7A1; ALDH2 |
|  | 4-Carboxyphenylglycine | 264.1013174 | 3.725681003 | 1.19595 | 1.78E-09 | 2.4919 | Pyrimidine Metabolism | 5'-nucleotidase;5'(3')-deoxyribonucleotidase, cytosolic type; Uridine-cytidine kinase 1; Uridine-cytidine kinase 2 | NT5E; NT5C; UCK1; UCK2 |
|  | Hydroxyphenylacetylglycine | 364.1995745 | 4.014362361 | 1.19962 | 0.00022026 | 0.87624 | Glycine and Serine Metabolism | - | - |
|  | Xanthurenic acid | 252.0492029 | 4.074093677 | 1.00377 | 1.98E-09 | 1.8491 | Tyramine metabolism | Glycine N-acyltransferase;Glycine N-acyltransferase-like protein 1 | GLYAT |
|  | L-Methionine S-oxide | 210.0420228 | 12.57534288 | 1.16876 | 0.00016385 | 0.36544 | Tryptophan Metabolism | HemK methyltransferase family member 1; Methyltransferase-like protein 2B; Uncharacterized methyltransferase WBSCR22 | HEMK1; METTL2B; WBSCR22 |
|  | Isovalerylsarcosine | 234.0625007 | 14.81337416 | 1.27427 | 1.81E-12 | 17.693 | Pyrimidine Metabolism | 5'-nucleotidase; Cytosolic 5'-nucleotidase 1B; Cytosolic 5'-nucleotidase 1A; 5'(3')-deoxyribonucleotidase, mitochondrial; Uridine-cytidine kinase 1 | NT5E; NT5C1B; NT5C1A; NT5M; UCK1 |
|  | Uric acid | 401.0732814 | 15.81989891 | 1.25091 | 3.49E-08 | 0.26936 | Amino Sugar Metabolism | Beta-hexosaminidase subunit beta; Beta-hexosaminidase subunit alpha; N-acetyl-D-glucosamine kinase | HEXB; HEXA; NAGK |
|  | Hydroxyacetone | 303.0318572 | 16.0836765 | 1.15202 | 0.0034368 | 0.79924 | Purine Metabolism | Xanthine dehydrogenase/oxidase | XDH |
|  | 2,8-Dihydroxyquinoline-beta-D-glucuronide | 336.074522 | 16.6402182 | 1.24548 | 2.23E-07 | 0.5799 | glycine, serine, and threonine metabolism | Retinol dehydrogenase 13 | RDH13 |
|  | O-Phospho-4-hydroxy-L-threonine | 251.0159494 | 16.75361232 | 1.21996 | 4.45E-06 | 0.36315 | - | UDP-glucuronosyltransferase 2B4 | UGT2B4 |
|  | Hypoxanthine | 341.0317021 | 17.31959417 | 1.03863 | 7.88E-09 | 1.7063 | - | Phosphoserine aminotransferase | PSAT1 |
|  | Creatine | 130.0621242 | 17.81266334 | 1.14461 | 0.00061971 | 1.52423 | Purine Metabolism | Xanthine dehydrogenase/oxidase; Hypoxanthine-guanine phosphoribosyltransferase; Purine nucleoside phosphorylase | XDH; HPRT1; PNP |
|  | Imidazole-4-acetaldehyde | 155.0463229 | 18.19761889 | 1.16015 | 0.0010023 | 1.2083 | Glycine and Serine Metabolism；Arginine and Proline Metabolism | Guanidinoacetate N-methyltransferase; Creatine kinase S-type, mitochondrial; Creatine kinase B-type; Creatine kinase U-type, mitochondrial; Creatine kinase M-type | GAMT; CKMT2; CKB; CKMT1A; CKM |
|  | Glycerol 3-phosphate | 171.0093263 | 18.45915672 | 1.15199 | 3.57E-07 | 2.5832 | Histidine Metabolism | 4-trimethylaminobutyraldehyde dehydrogenase; Aldehyde dehydrogenase, mitochondrial; Amiloride-sensitive amine oxidase [copper-containing] | ALDH9A1; ALDH2; ABP1 |
|  | Histamine | 301.0368094 | 18.55631762 | 1.0686 | 0.0083275 | 1.1372 | Phospholipid Biosynthesis；Glycerolipid Metabolism | Glycerol-3-phosphate dehydrogenase, mitochondrial; Glycerol kinase; Glycerol-3-phosphate acyltransferase 1, mitochondrial; Putative glycerol kinase 3; Glycerol-3-phosphate acyltransferase 3; Glycerol-3-phosphate acyltransferase 2, mitochondrial | GPD2; GK; GPAM; GK3P; AGPAT9; GPAT2 |
|  | Pyrophosphate | 300.0418249 | 18.60009533 | 1.20211 | 8.07E-05 | 0.6786 | Histidine Metabolism | Aromatic-L-amino-acid decarboxylase; Amiloride-sensitive amine oxidase [copper-containing]; Histamine N-methyltransferase; Histidine decarboxylase | DDC; ABP1; HNMT; HDC |
|  | L-Threonine | 186.0390178 | 18.65102394 | 1.20703 | 2.65E-05 | 0.58507 | Alanine Metabolism | Acetyl-coenzyme A synthetase, cytoplasmic; Fucose-1-phosphate guanylyltransferase; Alpha-aminoadipic semialdehyde dehydrogenase; Nicotinamide mononucleotide adenylyltransferase 1 | ACSS2; FPGT; ALDH7A1; NMNAT1 |
|  | N-Acetylgalactosamine 4-sulphate | 298.1139619 | 18.8156096 | 1.23726 | 1.12E-07 | 3.3656 | Glycine and Serine Metabolism | Threonine--tRNA ligase, cytoplasmic | TARS |
|  | Adenosine | 326.1120063 | 18.8297891 | 1.26387 | 1.04E-06 | 0.49808 | - | Hyaluronidase-1 | HYAL1 |
|  | L-Serine | 595.1155157 | 18.88261488 | 1.14412 | 5.48E-06 | 2.0294 | Purine Metabolism | 5'-nucleotidase; Adenosine kinase; Adenosylhomocysteinase | NT5E; ADK; AHCY |
|  | L-Glutamine | 145.0622488 | 19.02521056 | 1.2668 | 2.51E-10 | 1.77432 | Sphingolipid Metabolism；Glycine and Serine Metabolism；Methionine Metabolism | Serine--tRNA ligase, mitochondrial; Serine hydroxymethyltransferase, mitochondrial; Serine palmitoyltransferase 1; Phosphoserine phosphatase | SARS2; SHMT2; SPTLC1; PSPH |
|  | Glycerophosphoinositol | 333.0616882 | 19.10440731 | 1.06964 | 2.57E-07 | 1.7208 | Alanine, aspartate and glutamate metabolism | Glutamine--fructose-6-phosphate aminotransferase [isomerizing] 2; Kynurenine--oxoglutarate transaminase 1; Asparagine synthetase [glutamine-hydrolyzing]; Glutamine synthetase; Glycine N-acyltransferase-like protein 1 | GFPT2; CCBL1; ASNS; GLUL; GLYATL1 |
|  | Vinylacetylglycine | 648.2061421 | 19.44080408 | 1.07658 | 2.68E-06 | 1.6611 | - | Glycerophosphodiester phosphodiesterase 1; Glycerophosphoinositol inositolphosphodiesterase GDPD2 | GDE1; GDPD2 |
